# Iterative Selection of DNA Nanostructures for Cellular Uptake

**DOI:** 10.1002/adma.74852

**Published:** 2026-08-31

**Authors:** Anjali Rajwar, Lisa Eichhorn, Jakub Palacka, Sandra Ly, Erik Benson

**Affiliations:** ^1^ Science for Life Laboratory (SciLifeLab) Department of Microbiology Tumor and Cell Biology Karolinska Institutet Solna Sweden

**Keywords:** cellular uptake, DNA library, DNA nanotechnology, selection

## Abstract

DNA nanotechnology offers precise, biocompatible structures with strong potential for targeted drug delivery, yet current discovery approaches rely on testing individual designs, limiting exploration of structural diversity. In this study, we introduce an iterative selection strategy that screens large libraries of DNA nanostructures, each folded from a single‑stranded “structure genome” compatible with amplification and sequencing. Cellular internalization is used as the selection pressure: libraries are incubated with mammalian cells, internalized structures are recovered from lysates, and the process is iterated across multiple rounds in HEK293T and RAW264.7 cells. High‑throughput sequencing of recovered structure genomes reveals cell‑type‐specific enrichment patterns, enabling the identification of individual nanostructures with preferential uptake. Selected candidates were synthesized and evaluated as purified structures, confirming differential internalization by quantitative flow cytometry and microscopy. Turning DNA nanostructure discovery into a selection‑based process could enable high‐throughput exploration of structural diversity and provide an alternative route to identify nanostructures with cell‑specific uptake properties for biomedical applications.

## Graphical abstract

DNA nanostructures are promising cell targeting delivery vehicles for therapeutics, but the targeting behavior is not fully understood. In this study, libraries of DNA nanostructures that can be amplified and sequenced are combined with cellular uptake as a selection pressure to iteratively refine DNA structures taken up in cells to better understand what drives uptake in different cell types.

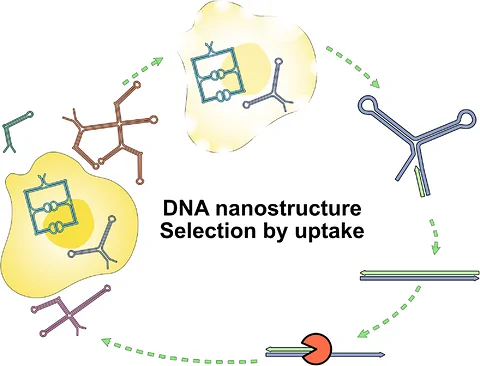

## 1 Introduction

A key goal of nanomedicine is to develop structures and particles with structural, mechanical, and functional properties allowing them to specifically interact with and be taken up by certain cells to deliver therapeutic payloads. DNA nanotechnology is a powerful method for producing self‐assembled nanostructures from oligonucleotides with fine control of size, shape, and mechanics, as well as offering the capacity to display patterns of other biomolecules such as proteins [1, 2, 3, 4, 5, 6]. This high degree of precision has allowed for the study of how design choices, such as size, geometry, and mechanical properties control the interaction with cells, tissues, and organisms. Previous studies have demonstrated the importance of structure–activity relationships in DNA nanostructure uptake. For example, Bastings et al. compared 11 distinct DNA origami designs, ranging from 50 to 400 nm in size, across multiple cell lines, and observed that larger, more compact structures were preferentially internalized relative to elongated, high–aspect‐ratio particles. Uptake rates, however, were shown to depend more strongly on cell type than particle geometry, with phagocytic cells such as dendritic cells exhibiting prolonged uptake without reaching saturation over 12 h [7].

Using the modular DNA brick strategy, researchers further engineered eight distinct DNA nanoparticle designs, including rectangular and tubular geometries of varying dimensions, for systemic siRNA delivery. Although all nanoparticles exhibited enhanced internalization compared to naked DNA, a small rectangular construct 6H x 96BP‐Rect (a 6‐helix‐wide, 96‐base‐pair‐long rectangle) achieved the highest uptake efficiency, reinforcing the significance of size and shape in cellular internalization [8]. Complementing these findings, another study reported that tetrahedral DNA nanostructures exhibited superior cellular internalization relative to other 3D geometries tested across both 2D and 3D cell culture models. This enhanced uptake was further confirmed in more complex biological contexts, including tumor spheroid invasion assays and epithelial barrier crossing in zebrafish models [9]. Together, multiple studies underscore the critical role of rational nanoparticle design, particularly shape and size, in optimizing cellular uptake for biomedical applications [1, 4, 6, 10, 11, 12, 13].

Despite these advances, a key limitation in exploring DNA nanostructure design space lies in the reliance on testing individual nanostructures one at a time, which makes it difficult to explore the vast design space available when shape, size, and mechanics can all be tuned independently. Iterative selection strategies offer a potential solution by enabling high‐throughput functional screening of large structural libraries within a single experimental context. Such approaches underlie the success of SELEX for aptamer discovery [14, 15, 16, 17], as well as DNA‐encoded chemical libraries for small‐molecule drug discovery [18, 19, 20].

However, unlike oligonucleotide‐based aptamers, most DNA nanostructures are assembled from multiple component strands via tile‐based [21] or origami methods [22]. When such assemblies are pooled, amplified, and sequenced, structural information is lost, and refolding libraries from amplified mixtures is unlikely, as component strands from different designs can misassemble. To overcome this, recent advances have explored rationally designed DNA [23] or RNA [24] nanostructures that fold from one strand, including constructs compatible with co‐transcriptional folding. In these designs, distal regions of the strand are programmed to form secondary and tertiary interactions, such as helices, junctions, and kissing loops. While linear DNA fragment libraries can be generated at scale, producing diverse DNA nanostructure libraries through this route remains challenging, as interacting domains must be both encoded within the same strand and spatially segregated to ensure correct folding. In this work, we introduce a new strategy for generating DNA nanostructure libraries based on the ligation of smaller preassembled nanostructure fragments of varying sizes and complexity. This approach yields highly diverse libraries in which each structure's overall architecture is defined by the specific combination of fragments. The resulting structures are diverse in shape yet remain traceable by sequencing. We apply this library in cellular selection experiments to investigate whether internalization by mammalian cells is enhanced by certain structural features. By performing iterative rounds of selection in HEK293T and RAW264.7 cells, followed by nanopore sequencing of internalized structures, we examine enrichment dynamics and compare results with Illumina sequencing for cross‐validation. Candidate structures identified from both cell types were further characterized to assess and compare their internalization efficiencies. We evaluated the cellular internalization of preferentially selected candidates across both selected cell lines as well as in the cell lines A549, MDA‐MB231, HeLa, and MCF‐7 that were not used for selection of candidates. Candidates enriched through selection exhibited the highest levels of internalization in their respective cell lines, whereas randomly selected, non‐enriched candidates showed comparatively greater uptake in nonselected cells. To further validate whether this selection strategy extends to a more clinically relevant setting, we generated a new library and performed five rounds of iterative selection in two cancer cell lines, MDA‐MB‐231 and A549, observing enrichment dynamics that matched or exceeded those seen after 10 rounds in HEK293T and RAW264.7 cells. Together, these findings demonstrate a new route to discover DNA nanostructures with cell‐targeting abilities by converting a design to a selection problem allowing the test of dramatically more DNA structures in a single‐pot experiment.

## 2 Results and Discussion

### 2.1 Design and Optimization of a DNA Nanostructure Library

We developed a library of 36 DNA nanostructure fragments that varied in size and complexity, each designed to assemble from one to four synthetic oligonucleotides (Table S1). The sequence of each fragment oligonucleotide was designed from a set of 20 nt sequences devoid of homopolymer stretches, balanced GC content, matching melting temperatures, and optimized to avoid cross‐interactions and secondary structure formation. The fragment set included a three‐way junction, two four‐way junctions, hairpins including kissing loops of various sizes, and linear fragments of different lengths (Figure 1A). For linear fragments, we varied the sizes ranging from 14 to 20 nt, while for bulged fragments we fixed the length at 20 nt and varied the bulge size from 1 to 5 nucleotides. In addition, an “amplification loop” hairpin was designed to provide primer‐binding sites for PCR and two embedded 10 nt unique molecular identifier (UMI) sequences for lineage tracing. All fragments were designed to feature AGT or ACT sticky‐end overhangs to facilitate ligation. The ligation was designed to be directional: linear fragments featured one AGT and one ACT overhang, while junction fragments featured one AGT overhang and multiple ACT overhangs. All structural hairpins featured AGT overhangs, whereas the amplification loop was designed exclusively with an ACT overhang. This is to drive each ligated DNA nanostructure to incorporate only a single amplification loop, preventing multiple amplification sites within a single assembly.

**FIGURE 1:**
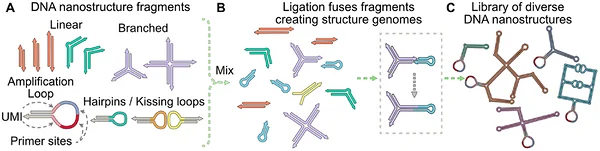
Schematic of generation and assembly of diverse DNA nanostructure library. (A) Small DNA nanostructure fragments of varying sizes and complexities: linear, bulged, branched junctions, hairpins, and amplification loop featuring primer‐binding sites and twin 10‐nt UMIs for lineage tracing. (B) The fragments are mixed and ligated via sticky ends to produce a diverse pool of DNA nanostructures (C). Many structures fold to form one single “genome strand”.

For library preparation, the complementary strands comprising each fragment type were mixed separately and annealed to fold into respective DNA nanostructure fragments (Figure 1B). The annealed fragments were then mixed and T4 ligation enzyme was added that joined the fragments via sticky‐end ligation of the designed overhangs generating a complex library of interconnected structural assemblies. This process created a heterogeneous pool of nanostructures formed by the “gluing” of the individual components, forming a long single‐stranded “structure genome” (Figure 1C). To refine the library and remove unbound or partially ligated fragments, the ligated pool was subjected to exonuclease III (Exo III) digestion. Exo III is a 3′→5′ exonuclease that specifically digests double‐stranded DNA (dsDNA) with free or recessed 3′ ends, while sparing DNA structures that lack accessible termini, such as circular fully ligated constructs. This selective degradation step effectively removed linear or incompletely ligated intermediates, which present free 3′ termini susceptible to Exo III attack. In contrast, fully ligated and topologically closed structures remained resistant to digestion, thereby enriching the pool for covalently closed and structurally complete nanostructures. The heterogeneity of the library and the impact of Exo III digestion were subsequently assessed by urea–PAGE and agarose gel electrophoresis. Following enzymatic treatment, several bands disappeared from the gel profiles, consistent with the selective removal of susceptible, non‐protected assemblies (Figure S1A). Finally, the structures from the ligated library containing the incorporated amplification loop were PCR amplified using primers specific to the loop, where one primer was produced with a 5’ phosphate modification for downstream enzymatic digestion. A gradient PCR across 50°C–60°C was used to find conditions that maintain library heterogeneity while minimizing nonspecific amplification. A no‐template control (NTC) showed no product at any temperature, confirming that all observed bands were template‐dependent. The successful PCR amplification of these structurally diverse templates generates a library of dsDNA structural genomes. The ExoIII‐treated library (Lib+ExoIII) amplified at all the tested temperatures; however, lower temperatures (50°C–55°C) yielded distinct low molecular weight bands, whereas higher temperatures (58°C–60°C) produced complex, smeared high molecular weight profiles, reflecting broader structural diversity (Figure S1B). Based on these observations, 60°C was selected as the optimal annealing temperature, as it yielded the most heterogeneous amplification profile. We next compared amplification between the undigested control library (Ctrl) and the Lib+ExoIII at 55°C, 58°C, and 60°C. The Ctrl showed amplification at all temperatures but yielded fewer high–molecular‐weight products whereas Lib+ExoIII produced more high–molecular‐weight bands, indicating more heterogeneity in the Lib+ExoIII amplified sample. In both cases, amplification efficiency and apparent product complexity increased with annealing temperature, with the most diverse profiles at 60°C (Figure S1C).

Following amplification, the library was purified to remove unincorporated primers, smaller fragments, and other reaction remnants resulting in the refined pool of assembled nanostructures. Next, this library was sequenced that provided insights into the structural composition and complexity of the initial library, effectively profiling the initial mix of nanostructures. For subsequent cell selection experiments, the purified initial library was subjected to lambda (*λ*) exonuclease digestion. This enzymatic treatment selectively degrades strands with 5’ phosphate included in one of the PCR primers, rendering the nanostructures single‐stranded. The resulting single‐stranded structures were then subjected to a refolding process in a ligation buffer to refold them into their native conformations and subsequently used for cell selection assays. This structure library was imaged by atomic force microscopy (AFM), revealing numerous DNA nanostructures including some circular assemblies that may represent structures containing kissing loops (Figure S1D).

### 2.2 Selection Through Repeated Cellular Uptake Experiments

To investigate the structure–activity relationship and determine whether specific DNA nanostructures exhibit preferential cellular internalization, we utilized the structure library for cell selection experiments. We chose two cell models that are known to take up DNA: HEK293T, a human embryonic kidney cell line often used for transfection, and RAW264.7, a mouse macrophage‐like cell line. A total of 2 µg of the DNA nanostructure library was separately incubated in HEK293T and RAW264.7 cells for 4 h to facilitate uptake. The concentration of library was kept constant at 2 µg across different rounds of selection in both the cell lines. Non‐treated cells were considered as negative control and were used as a comparative control to evaluate potential genomic or sub‐cellular contamination. By analyzing their amplification profiles alongside those of the treated samples (cells exposed to nanostructures), we were able to verify that the sequences detected in downstream PCR originated from genuinely internalized nanostructures rather than background cellular material or processing artifacts. Following incubation, the cells were washed to reduce surface‐bound nanostructures, allowing for the analysis of primarily internalized DNA. The cells were then lysed using RIPA lysis buffer and the resulting lysate was subjected to bead‐based DNA purification to recover the DNA structures. Both treated and non‐treated (control) purified lysate was subjected to agarose gel electrophoresis that revealed high molecular DNA bands that likely correspond to either genomic or some organellar DNA. Next, upon PCR amplification of these lysates using primers targeting the amplification loop, a faint smear together with discrete bands appeared only in the treated sample that had been exposed to the DNA nanostructure library, whereas the control lysate yielded no detectable amplification product (Figure S2A). In order to verify that amplification was specific to our library, we performed in silico PCR using UCSC genome browser BLAT tool against the human reference genome (GRCh38). No significant hits were identified, indicating that our primers do not nonspecifically bind to host genomic DNA [25]. We next tested PCR amplification for 10, 15, 20, and 25 cycles to determine an optimal cycle number that maximized yield while minimizing nonspecific amplification. Although band intensity increased with cycle number, 15 cycles produced sufficient amplification and was therefore used for all subsequent lysate amplification experiments (Figure S2B). The amplified nanostructures were first purified using AMPure XP beads and then treated with lambda exonuclease to generate single‐stranded DNA (ssDNA), followed by refolding in ligation buffer to reconstitute structured conformations. The refolded nanostructures were then reintroduced into their respective cell lines for the next round of selection. This iterative workflow of selection, comprising lysate purification, PCR amplification, purification, lambda exonuclease digestion, refolding, and re‐incubation with the target cells, was repeated for a total of 10 cycles (Figure 2A). A representative agarose gel illustrating the sequential processing steps for the Round‐6 lysate prior to Round‐7 selection is shown in (Figure S2C).

**FIGURE 2:**
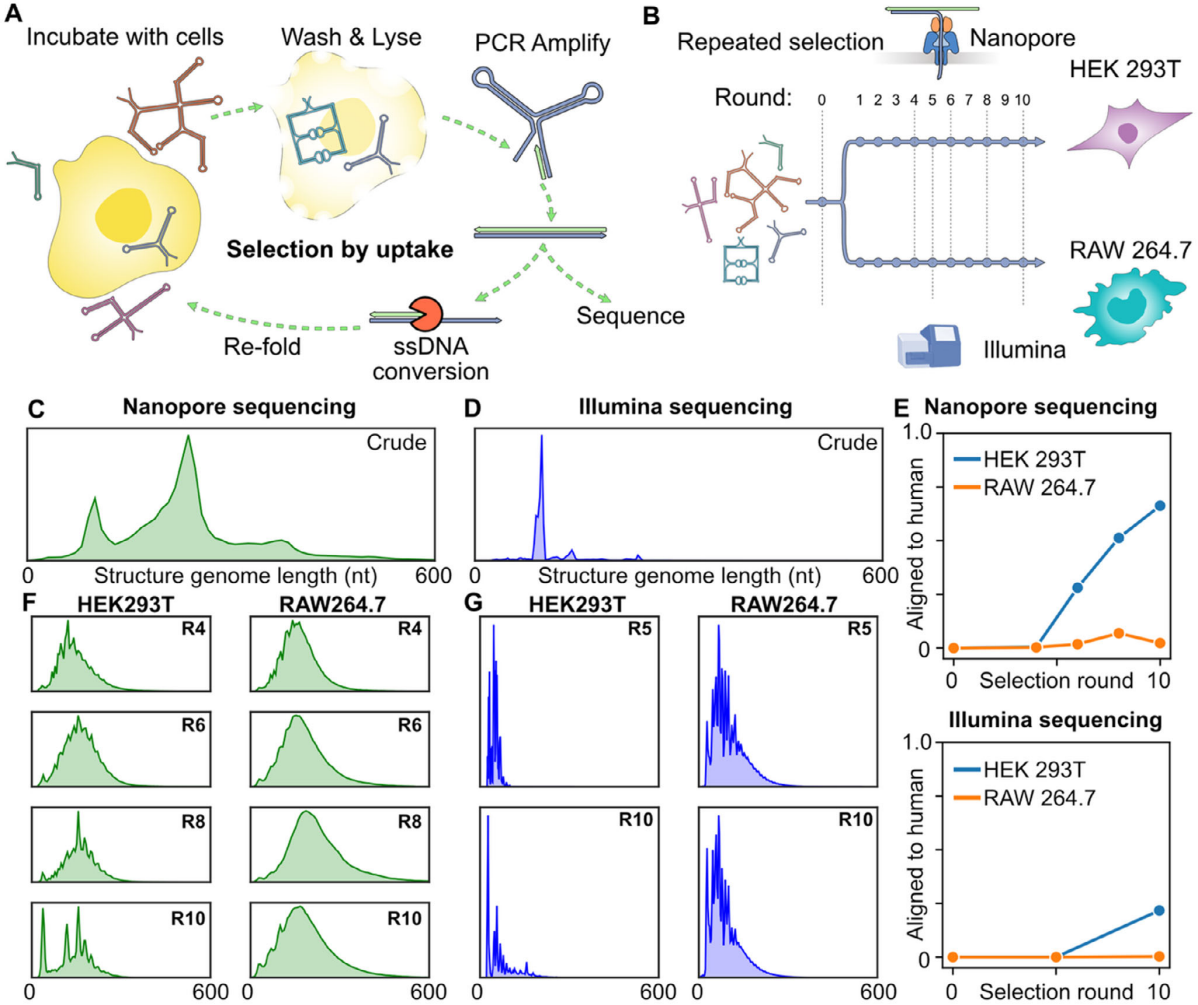
Selection of DNA nanostructures through cellular uptake (A) Schematic of uptake‐mediated selection workflow for DNA nanostructures. DNA nanostructure library is incubated with cells followed by washing and lysis to recover internalized nanostructures. Structures are then PCR‐amplified followed by ssDNA conversion using lambda exonuclease and refolded for the next round of selection. (B) The process is repeated and combined with high‐throughput sequencing to study the structural preferences for cellular uptake. The initial DNA nanostructure library was sequenced on (C) Oxford Nanopore and (D) Illumina sequencing platforms. (E) Fraction of reads aligning to the human genome, indicating contamination from cellular DNA. (F, G) Structure genome length distribution over selection rounds in both cell lines using two sequencing methods after filtering out human genomic DNA reads.

To ensure that enrichment patterns are indeed due to cellular internalization rather than artifacts arising from PCR bias or preferential retention during purification, we performed an empty selection experiment in which the processed initial library was carried through 10 rounds of amplification and purification in the absence of any cellular selection step. Through this iterative enrichment process, we aimed to identify nanostructure candidates that consistently exhibited preferential internalization by HEK293T or RAW264.7 cells rather than any PCR amplification bias, thereby revealing insights into the structural features that drive cellular uptake.

After each round, aliquots of the amplified and purified dsDNA structure genomes were saved for later high‐throughput sequencing to monitor the selection process (Figure 2B). Oxford Nanopore sequencing (ONT) was used as it allows for rapid ligation sequencing with long read lengths at a relatively small scale, although with a lower read quality compared to other sequencing platforms, especially at the ends of the reads (where the UMI regions are located). The initial library as well as the selected libraries from rounds 4, 6, 8, and 10 in both cell lines were sequenced on flongle flow cells using a minion device, resulting in between 300k and 900k reads per library (Figure 2B).

To validate sequencing results from ONT the initial library as well as the selected libraries from rounds 5 to 10 in each cell line were re‐sequenced using the Illumina sequencing platform. Illumina sequencing has an error rate that is typically an order of magnitude lower, with the highest read quality at the ends, and offers flow cells with significantly more reads than ONT. However, the longest commercially available Illumina read is 600 nt, and the system has a strong preference for clustering and reading shorter fragments in pools with mixed read distribution [26]. We prepared the libraries for Illumina sequencing by adding read primers and adapters via PCR. This resulted in between 6.2 and 8.7 M reads per library (Figure 2B). The read length distribution of the corresponding libraries is markedly different in the two sequencing methods. In the initial library, ONT sequencing shows a smooth distribution with peaks at 100, 240, and 370 nt; in contrast, Illumina sequencing shows an uneven distribution with peaks at 100, 140, and 240 nt where the peak at 100 nt dominates the sequencing (Figure 2C,D).

A concern when amplifying DNA structures from cells is that we may also accidentally amplify genomic DNA from the cells. To detect potential genomic contamination these reads were aligned to the reference human genome GRCh38 [27]. As expected, we got no alignments in the initial libraries (R‐0), as no human DNA should be present. This was confirmed by both nanopore and Illumina sequencing data for R‐0 (Figure 2E). The fraction of aligned sequences increases steadily in the HEK293T data sequenced by nanopore, up to around 62% of the reads in Round 10 (R‐10). The fraction aligned in the corresponding Illumina sequencing is lower, reaching 22% in R‐10 of selection in HEK293T; this could be due to the size preference of Illumina sequencing disfavoring longer fragments of genetic origin. In contrast, the fraction of reads mapping to the human genome in the RAW264.7 cell line remained below 7% in all rounds and both sequencing methods. This may be due to the more promiscuous macrophage cells taking up DNA more effectively, leading to more target DNA in the cell lysate and less unspecific amplification. The data from RAW264.7 was also aligned to a mouse reference genome as validation, yielding similar results [28] (Figure S3). Still, even though the sequencing round was mostly contaminated with human DNA we still got 80k reads for analysis of DNA nanostructure uptake in cells.

After filtering out reads not from DNA nanostructures, the preference for structure sizes in the two cell lines can be seen in the change of the distribution of genome lengths. In ONT sequencing of the cell uptake in HEK293T cells a transition can be seen from the smooth distribution in the initial library to a shorter average size with several pronounced read length spikes (Figure 2F). A similar effect can be seen in the Illumina sequencing, although with a stronger preference for shorter sequences (Figure 2G). Interestingly, the selection in RAW264.7 cells shows a more modest change in length distribution in both sequencing methods compared to the initial distribution, further indicating that macrophage cells promiscuously uptake DNA structures with little size preference.

### 2.3 Structural Preferences in Cellular Uptake

The sequencing data derived from the initial library and evolved library pool after successive rounds of selection allow for the analysis of both general structure characteristics that increase cell uptake and the identification of individual nanostructures that are evolutionarily favored. Each DNA nanostructure genome is a combination of ligated fragments, terminated at either end by 10‐nucleotide UMI regions and the primer binding sites from the amplification loop (Figure 3A). By decoding the genome fragment composition, we can understand what type of structure features lead to preferential uptake in certain cell types. Although nanopore sequencing has a higher error rate, it is possible to reconstruct the fragment composition of the structure genomes using the tool concept that we recently developed [29]. Analysis of the initial library revealed that most structures comprise between 3 and 13 unique fragment types (Figure S4A). The library exhibited high diversity with the majority of fragment combinations represented by only one or a few reads; however, a subset of fragment combinations are significantly more common, occurring in hundreds of reads (Figure S4B). By calculating the composition of structures and relative abundance of fragments in the initial library and comparing it to later sequencing rounds, we can calculate the change in abundance of each fragment type and use it to infer evolutionary fitness benefits of structures including certain fragments. This revealed a clear preference for linear fragments in both cell lines as well as a strong negative selection for kissing loop segments (Figure 3B). The relative changes were larger in the HEK293T selection experiments than in the RAW264.7, again indicating that the macrophage cell line is more promiscuous in uptake. We further analyzed the changes in fragment combination frequencies between the initial library and the selected sequencing data, identifying combinations that showed the highest enrichment or depletion across 10 rounds of selection. Notably, certain fragment combinations exhibited more than 100‐fold increases or decreases in abundance. The comparison between the two cell lines revealed that while some fragment combinations were enriched in both, others were unique to each respective cell line, suggesting cell type‐specific selection pressures (Figure S5). The analysis also allowed us to look at the ordering of fragments within the structure genomes; surprisingly, many structures did not contain the theoretically expected ordering of fragments, indicating that ligation may occur through alternative or noncanonical assembly rather than strictly following the intended design (Figure S6). One possible explanation is that off‐target ligation of the non‐palindromic toehold could lead to the ligation of non‐hybridized fragment oligonucleotides or the incorporation of multiple amplification loops, creating a higher‐than‐expected diversity of structures in the library.

**FIGURE 3:**
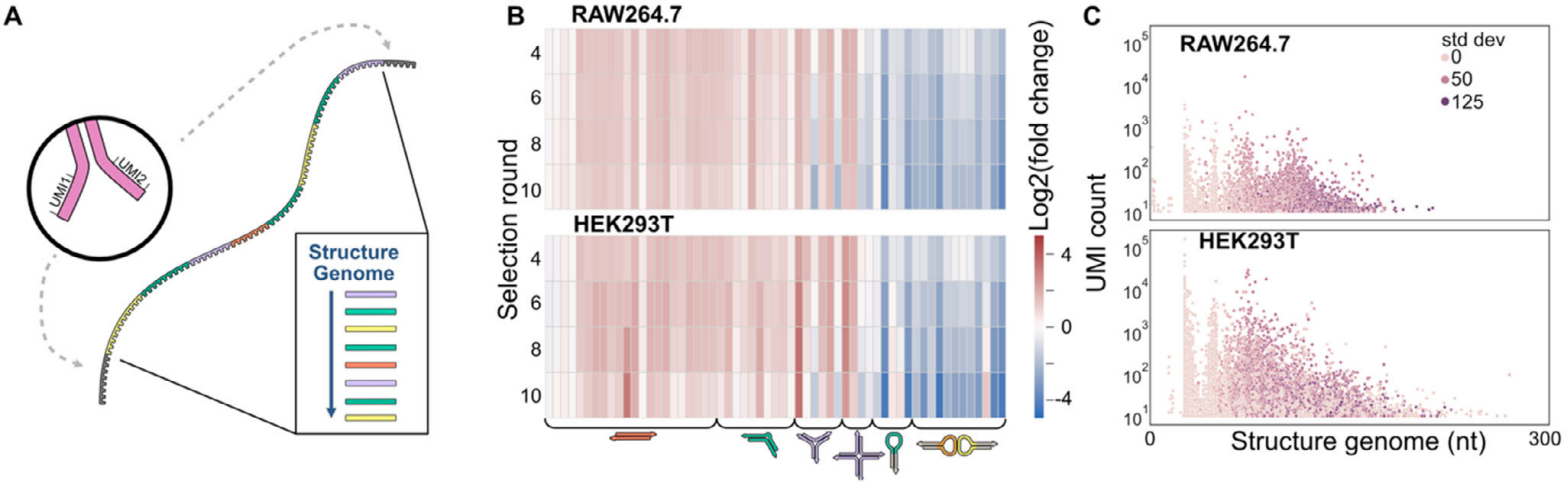
Analysis of sequenced DNA nanostructure genomes. (A) Each structure genome consists of ligated constituent fragments and is terminated at both ends with a 10 nt unique molecular identifier (UMI). (B) ONT data showing change in relative abundance of structure fragments compared to the initial library in two cell lines, filtered to remove reads mapping to the human genome. (C) Illumina data showing distribution of abundance, genome size (excluding primer sites), and length variability of UMI‐labeled structure genomes after 10 rounds of selection in two cell lines.

Each structure genome is labeled with one 10 nt UMI at either end of the genome, together these two UMIs result in a 20 nt barcode for each unique structure formed at the initial library generation. The underlying diversity of 4^20^ » 10^12^ means that it is unlikely for several structures to get identical barcodes, a histogram of UMI occurrences in the initial library showed that 86.4% and 99.7% of reads had unique UMIs in Illumina and ONT sequencing data, respectively (Figure S7). The analysis of structure UMIs requires the comparison and identification of identical 20 nt segments between reads where read errors are likely to lead to misidentification. ONT sequencing has an error rate that can be above 10% in the start of reads making the accurate determination of UMI identity somewhat difficult. In comparison, Illumina sequencing typically offers an error rate below 0.1% in the start of reads, making it suitable for the identification of reoccurring UMIs. Plotting the abundance of individual UMIs together with the corresponding genome length after 10 rounds of selection revealed thousands of individual DNA nanostructures that have increased in number. This effect is clearer in HEK293T cells with 238 UMIs appearing more than 1000 times in sequencing compared to just 11 UMIs in the RAW264.7 selection experiments, indicating a more stringent selection process in the HEK293T cells (Figure 3C). The difference in genome length in structures with identical UMIs is illustrated by plotting the standard deviation of the extracted genomes, for many UMIs the standard deviation is 0 but for some it is significant, indicating that structure genomes have experienced divergent mutations during the selection process. Analysis of structure genomes that share the same UMI revealed a median sequence identity of 99.1% in Round 10 from the HEK293T selection, indicating that the UMI can effectively label and track genomes throughout the selection process (Figure S8).

In our control experiment where we performed 10 rounds of “empty” selection, the samples from Round 5 (R‐5) and Round 10 (R‐10) were sequenced using the ONT platform. This data revealed no strong preference for any UMI, in the sequencing data after 10 rounds the top UMI occurred only in 0.02% of reads and around 97% of reads are labeled with unique UMIs. The empty selection resulted in a relatively smooth length distribution with higher average genome lengths than either of the cell selections (Figure S9A). Similar to the cell selection, loops were negatively selected for, although the changes were smaller in the empty selection (Figure S9B).

### 2.4 Identification and Production of DNA Nanostructure Candidates From Sequencing Data

#### 2.4.1 Selection From Nanopore Data

Nanopore sequencing showed enrichment of some candidate sequences that were progressively enriched across successive selection rounds. This prompted us to further investigate these candidates to understand the basis of their preferential accumulation. At this point, it remained uncertain whether this enrichment reflected genuine cellular selection pressures or resulted from stochastic events or methodological biases inherent to the process. In our initial analysis, we prioritized sequences longer than 200 nucleotides and further filtered candidates with high UMI counts after selection. Based on their strong enrichment profile and consistent growth across selection rounds, we initially selected four candidate structure genomes of lengths 208 bp‐H5A, 283 bp‐H5B, 375 bp‐H5C, and 485 bp‐H5D from HEK293T Round‐5 nanopore data. These candidates exhibited distinct enrichment dynamics over the 10‐round selection cycle. H5A and H5C showed sustained exponential enrichment through Round 10, with H5A displaying the most robust increase. In contrast, H5B and H5D stagnated following Round 8 (Figure S10A).

To validate these enrichment profiles and produce these structures individually for further testing, we designed structure‐specific primers targeting UMI regions at the start and end of the structure genomes of interest, with the reverse primers labeled with a Cy5 fluorophore (Table S2). These primers allowed us to selectively amplify each candidate structure from purified lysates collected across different selection rounds to further validate their enrichment profile. Using PCR‐amplified and purified Round‐5 lysate as the template, we tested different annealing temperatures ranging from 45^°^C to 50^°^C based on the annealing temperature of primer pairs and successfully amplified all four candidates, confirming their enrichment. We imaged the gel in both SYBR Safe (Figure S10B) and Cy5 (Figure S10C) channels to verify specific amplification and incorporation of the Cy5‐labeled primer. We observed a single discrete band for each candidate, without any nonspecific amplification in both channels and minor signal from unincorporated primers at the gel bottom in the Cy5 channel. We next purified these PCR‐amplified candidates using AMPure beads and the SYBR Safe channel revealed discrete bands for the purified structures (Figure S10D), while the Cy5 channel (Figure S10E) confirmed the removal of free, unincorporated primers from the gel bottom, ensuring high purity for further processing.

These structures were selected before we were aware of and filtered out structure genomes that aligned with human genomic DNA. The subsequent analysis revealed that these highly enriched sequences comprised a mixture of genomic fragments and synthetic elements originating from the initial library. We believe that these structure–genome chimeras arise during the PCR amplification step between selection rounds. One possible mechanism is that partially degraded DNA nanostructures containing unexpected 3′ termini act as primers by hybridizing to genomic DNA fragments present in the recovered cell lysate. Subsequent extension by DNA polymerase can generate chimeric molecules containing both nanostructure‐derived and genomic DNA sequences, which are then amplified in later PCR cycles and propagated through the selection process. We next determined the genomic content of these highly enriched candidates; we mapped these candidates to the human reference genome (GRCh38) using the blast‐like alignment tool (BLAT). Alignment analysis revealed that all analyzed candidates produced multiple alignment hits with variable score distributions. The top five hits for each candidate, along with their detailed alignment maps, are presented in (Figure S11A). Notably, across all analyzed candidates, alignments were localized to the 5' or internal segments of the query, with no significant alignment observed at the 3' terminus (Figure S11B).

**TABLE 1:** Reaction setup for exonuclease digestion of annealed and ligated DNA library.

Samples name	Sample vol. (µL)	Exo‐III (µL)	MilliQ (µL)	Total vol (µL)
Ligated mix	95	5	0	100
Negative control	45	0	5	50

To further investigate the extent of genomic contamination, we performed PCR amplification of each candidate from the initial (preselection) library R‐0, empty selection library R‐5 (Figure S12A) and R‐10 (Figure S12B), and non‐treated purified cell lysate (Figure S12C). We observed that the initial library R0 showed all candidates with a faint H5D signal; R5 showed strong signals except for H5D, while R10 showed reduced H5C and no H5D. The HEK293T control showed a signal for H5A and a faint H5B. We performed an initial series of cell‑uptake assays to characterize the intracellular behavior of each individual structure. Although later analysis showed that the H5A and H5B candidates contain genomic fragments, their pronounced enrichment warranted further study. Using templates from selection Round 5, we monitored the uptake of H5A, H5B, H5C, and H5D. Cy5 fluorescence was substantially higher in treated cells than in untreated controls, with H5B and H5C showing more diffusive and homogenous fluorescence compared to H5A and H5D (Figure S13A). By Round 10 there was a significant shift with H5B showing the brightest signal compared among all the samples. The initial library showed no visible fluorescence signal compared to the R‐10 library, which has some fluorescence signal but weaker than H5B highlighting the significance of enrichment of library and selected candidates in the cellular uptake (Figure S13B). To exclude the possibility that the fluorescence signal originated from the Cy5‐labeled primer alone rather than from amplified products, we performed a primer‐only control in which the forward primer and Cy5‐labeled backward primer were mixed and incubated with the cells. These samples showed minimal fluorescence, further confirming that the observed signal arises from the corresponding amplified structures (Figure S14). Subsequently, the candidates were evaluated for internalization in both HEK293T and RAW cells to assess potential preferences for sequences selected in a specific cellular context.

#### 2.4.2 Selection From Illumina Data

We next selected candidate sequences from both RAW264.7 and HEK293T datasets generated by Illumina sequencing, given the higher read accuracy of Illumina data. Candidates were chosen primarily from Round 10 samples of both cell lines. At this stage, long dsDNA fragments (up to 600 bp) corresponding to these candidates were synthesized as single contiguous constructs by ANSA Biotechnology. To enable downstream amplification and fluorescence‐based tracking, primer‐binding sites were incorporated at both the 5′ and 3′ termini of each construct. This design enabled selective PCR amplification using Cy5‐labeled primers for subsequent cellular uptake assays. Candidate selection was designed to capture enrichment patterns from both cell lines and multiple selection rounds. Since earlier candidates had been derived predominantly from HEK293T selections, we expanded our analysis to include RAW264.7–derived sequences. Four candidates were selected from RAW264.7 Round 10 (R10A, R10B, R10C, and R10D), based on their high UMI counts and absence of genomic DNA. In parallel, four candidates from HEK293T Round 10 Illumina data were selected and labeled H10A, H10B, H10C, and H10D using the same selection criteria.

In addition, we applied two strategies to select control candidates for experimental comparison. First, we selected four candidates that appeared in the Illumina sequencing data of the initial library but were completely absent from Illumina sequencing data of the selected libraries; these candidates were referred to as unselected control A–D. Secondly, we applied random selection from Illumina data of the initial library to find 9 candidates of different lengths, without any consideration of their enrichment or depletion status and these were referred to as Random 1–9. However, subsequent analysis revealed that most of the randomly selected candidates were likewise absent from the later rounds of selection and therefore represented non‐enriched structures similar to the unselected controls. Nevertheless, the two groups were retained as distinct categories to reflect their different selection criteria and to provide a broader set of non‐enriched reference structures for comparison with the enriched candidates. Sequence identities and corresponding UMI counts for all candidates are provided in (Table S3).

The integrity and purity of the synthesized fragments were first verified by gel electrophoresis, which confirmed the presence of a single discrete population for each construct (Figure S15A). These fragments were subsequently used as templates for PCR amplification. The amplification of ANSA‐synthesized candidates selected from HEK293T and RAW264.7 selections proceeded robustly and yielded single discrete bands after PCR purification, lambda exonuclease digestion, and refolding (Figure S15B&C). However, the initial amplification of random and unselected control sequences proved more challenging. Although these control templates appeared as single populations prior to amplification, PCR reactions frequently produced multiple bands indicating nonspecific amplification for these constructs (Figure S16A–C). We tried optimizing the PCR conditions and observed that elevated primer and template concentrations might have contributed to nonspecific amplification. Accordingly, the initial denaturation time was extended from 3 to 5 min, primer concentration was reduced significantly from 0.4 to 0.1 µM, and template concentration was lowered from 0.1 to 0.01 nM. These adjustments effectively eliminated nonspecific products and enabled clean amplification of all candidate and control sequences (Figure S16D). Following optimization, a total of 14 candidates were selected for subsequent cellular uptake studies. These included enriched sequences derived from both HEK293T and RAW264.7 selections, as well as non‐enriched random sequences serving as negative controls. From the HEK293T selection, candidates H10A, H10B, H10C, and H10D were chosen, while from the RAW cell Round 10 selection, candidates R10A, R10B, R10C, and R10D were selected. In addition, non‐enriched random sequences (Random1, Random4, and Random6) and the unselected control‐C (US‐C) were included as negative controls. The H5B and H5D candidates from the initial screen were also synthesized independently (ANSA) and included in the study. The sequence information of all the finalized candidates are provided in (Table S4). We also verified the selected candidates before cell stimulation via gel electrophoresis and observed a single predominant population, as shown in Figure S17. Fragment composition analysis of the selected candidates revealed that most structures comprise multiple fragments derived from the initial library. In contrast, H5B and H5D contained only a single library‐derived fragment in addition to the primer sequences and were predominantly composed of genomic DNA‐derived regions (Figure S18). The secondary structure prediction of the finalized candidates performed using mFold [30] depicts a diverse structural spectrum where the most stable conformations of selected candidates displayed higher prevalence of loops whereas the randomly selected candidates displayed distinctly longer ds stems (Figure S19). The thermodynamic stability and other structural characteristics of these candidates were evaluated based on mFold prediction data with results summarized in Table S5. In order to better understand the 3D conformation of these structures, we performed secondary structure prediction using NUPACK [31], resulting in the predicted base‐pairing at thermodynamic equilibrium. This prediction was used to create an initial conformation in the coarse‐grained molecular dynamics tool oxDNA [32] as well as potentials keeping predicted base pairs together; after initial relaxation, simulations were run at 20°C with 1 M Na and visualized using oxView [33] (Figure S20), displaying a mixed set of structures with several junctions.

### 2.5 Cellular Profiling of Candidates by Flow Cytometry and Microscopy

Following the final selection of candidate structures from both cell lines, we assessed and compared their cellular internalization as purified individual nanostructures. We have a total of 14 candidates, and each candidate was amplified with fluorophore‐labeled primers, purified, subjected to lambda exonuclease digestion, and refolded according to the protocol described above. The refolded structures were incubated with cells at a final concentration of 25 nM for 1 h and analyzed using flow cytometry (Figure 4A). Given that these candidates were evaluated as purified individual structures, internalization was expected to occur more efficiently than in pooled library conditions.

**FIGURE 4:**
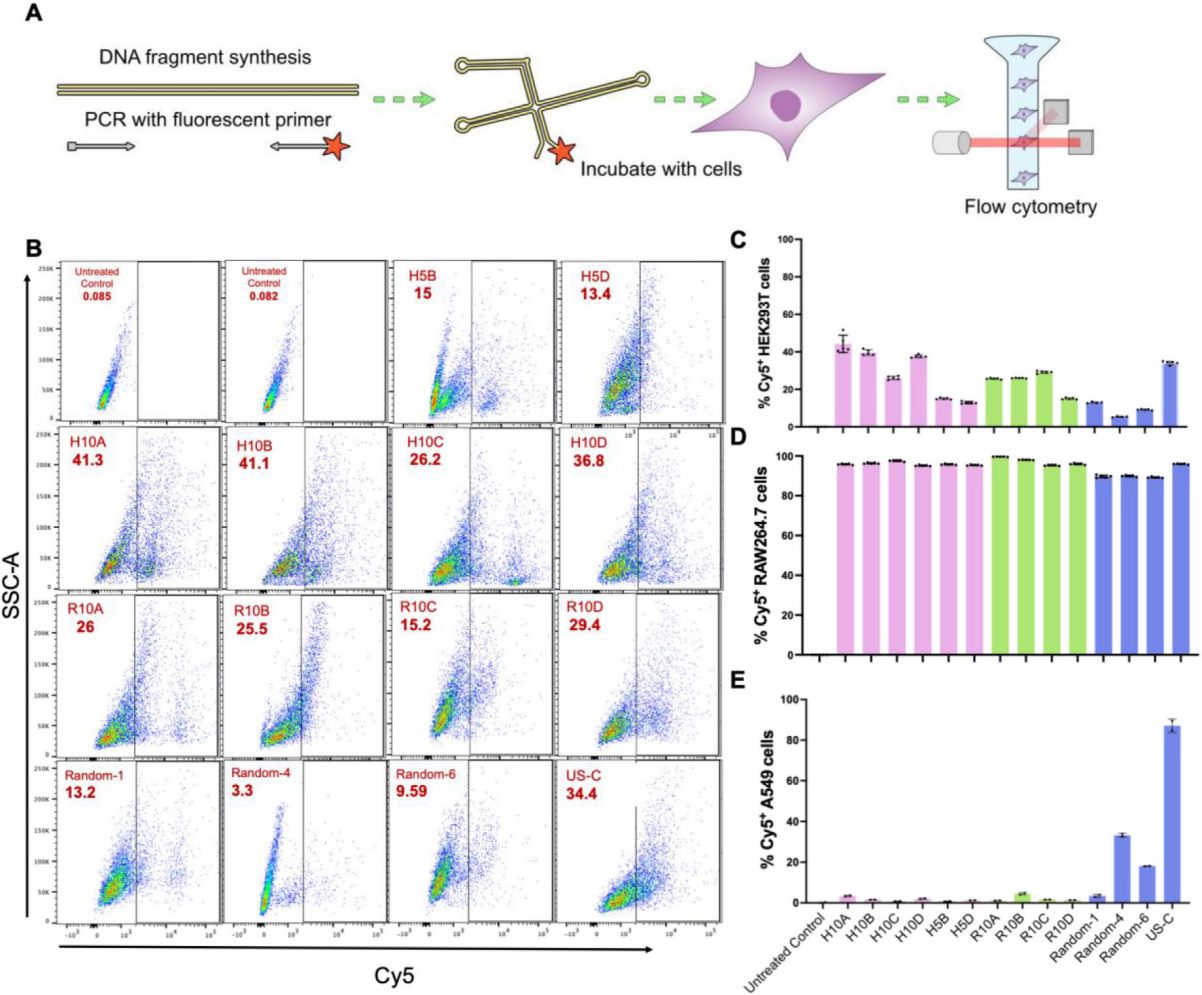
Quantification of DNA nanostructure internalization via flow cytometry. (A) Schematic illustrating the flow cytometry workflow where each candidate nanostructure was amplified using Cy5‐labeled primers, refolded and incubated with cells, and subsequently analyzed using a flow cytometer to quantify cellular uptake. (B) Representative flow cytometry scatter plots of selected candidates showing Cy5+ cells in HEK293T cells. (C) Quantification of Cy5+ HEK293T cells as a percentage of total cells in each sample group. (D) Quantification of Cy5+ RAW264.7 cells as a percentage of total cells in each sample group. (E) Quantification of Cy5+ A549 cells as a percentage of total cells in each sample group. Each sample was measured in quintuplicate (*n* = 5), and the experiment was performed independently twice (*N* = 2). Error bars indicate mean ± SD.

We performed flow cytometry with the finalized candidates to quantitatively evaluate DNA uptake at single‐cell resolution. This method provides a broader quantitative assessment by recording multiple events per sample allowing robust statistical comparison of uptake efficiencies across different treatment conditions. For each sample, 10 000 events were acquired, and all measurements were performed in triplicate or quadruplicate. Cy5 fluorescence was detected in the APC channel.

In HEK293T cells, the untreated control population remained entirely confined to the left region of the APC histogram, indicating negligible background signal. In contrast, all treated samples exhibited a rightward shift, consistent with cellular internalization of DNA nanostructures. (Figure 4B). Among the tested candidates, H10A and H10B showed the largest Cy5‐positive cell populations, followed by H10D and the US‐C (Figure 4C). Notably, candidates derived from the enriched cell line demonstrated higher levels of internalization compared with those selected from the RAW cell line. Interestingly, US‐C exhibited a substantially larger Cy5‐positive cell population than most randomly selected candidates, highlighting an inherent limitation of the selection strategy whereby potentially effective candidates may not be enriched or identified.

In the RAW264.7 macrophage‐like cells, treatment induced a pronounced rightward shift of the entire cell population, reflecting the intrinsically high and promiscuous uptake capacity of this cell line. Utilizing this gating strategy, > 90% of the population was Cy5‐positive (Figure 4D). To better resolve differences among candidates, a Cy_5_^high^ gate was applied to identify strongly positive cells. Within this gated population, R10B yielded the largest Cy5‐positive cell population, followed by US‐C and R10A, whereas randomly selected candidates exhibited the smallest proportion of Cy5‐positive cells (Figure S21).

To study the uptake behavior of structures in a cell line not used in selection, we used the human lung cancer cell line A549. Compared to the other cell lines, the overall DNA uptake was low. In the flow histogram for selected candidates the majority of the cell population is fixed toward the left and very few cells move toward the right, whereas control candidates induced a greater rightward shift (Figure S22). Unselected control‐C exhibited the highest proportion, followed by Random‐4 and Random‐6. All remaining candidates resulted in fewer than 10% Cy5‐positive cells, indicating limited internalization in this cell line (Figure 4E). In addition to quantifying the percentage of Cy5‐positive cells, we analyzed the mean fluorescence intensity (MFI) of each candidate to assess the extent of nanostructure uptake on a per‐cell basis (Figure S23). In HEK293T cells, H10C exhibited the highest MFI among all tested candidates, despite not producing the highest percentage of Cy5‐positive cells. This suggests that while H10C was internalized by a smaller fraction of the cell population, cells that did internalize the structure accumulated higher levels of fluorescence (Figure S23A).

In RAW264.7 cells, the highest MFI values were observed for R10B and unselected structure C, indicating efficient uptake or intracellular accumulation of these structures (Figure S23B). In A549 cells, unselected structure C also displayed the highest MFI, consistent with its elevated percentage of Cy5‐positive cells (Figure S23C).

We next employed laser scanning confocal microscopy to determine whether the DNA nanostructures were merely surface‐bound or truly internalized. Optical sectioning was performed for each sample and only the central z‐slices were used to generate representative images, thereby minimizing contributions from surface‐associated fluorescence. All samples were imaged under identical acquisition settings to permit comparison of fluorescence intensity across cell types and candidates.

In HEK293T cells, uptake was highly heterogeneous: a subset of cells exhibited intense fluorescence throughout the cytoplasm and nucleus, whereas others displayed sparse, punctate signals. Candidate H10A showed some nuclear localization, while H10B, H10C, and H10D were confined predominantly to the cytosol; notably, H10C and H10D produced markedly weaker fluorescence than H10A and H10B. H5B and H5D showed a more homogenous and diffusive cytosolic signal (Figure 5A). Some of the RAW cell‐selected candidates, like R10B seemed to show nuclear signal. The signal from R10B was only nuclear; however, it looked like the bleed‐through of the Hoechst signal. But all the images were acquired under sequential and identical settings, so this nuclear signal could indeed be due to the candidate. R10C and R10D showed heterogeneous signal like the H10 candidates (Figure 5B). Surprisingly, the randomly selected control candidates showed hardly any signal (Figure 5C).

**FIGURE 5:**
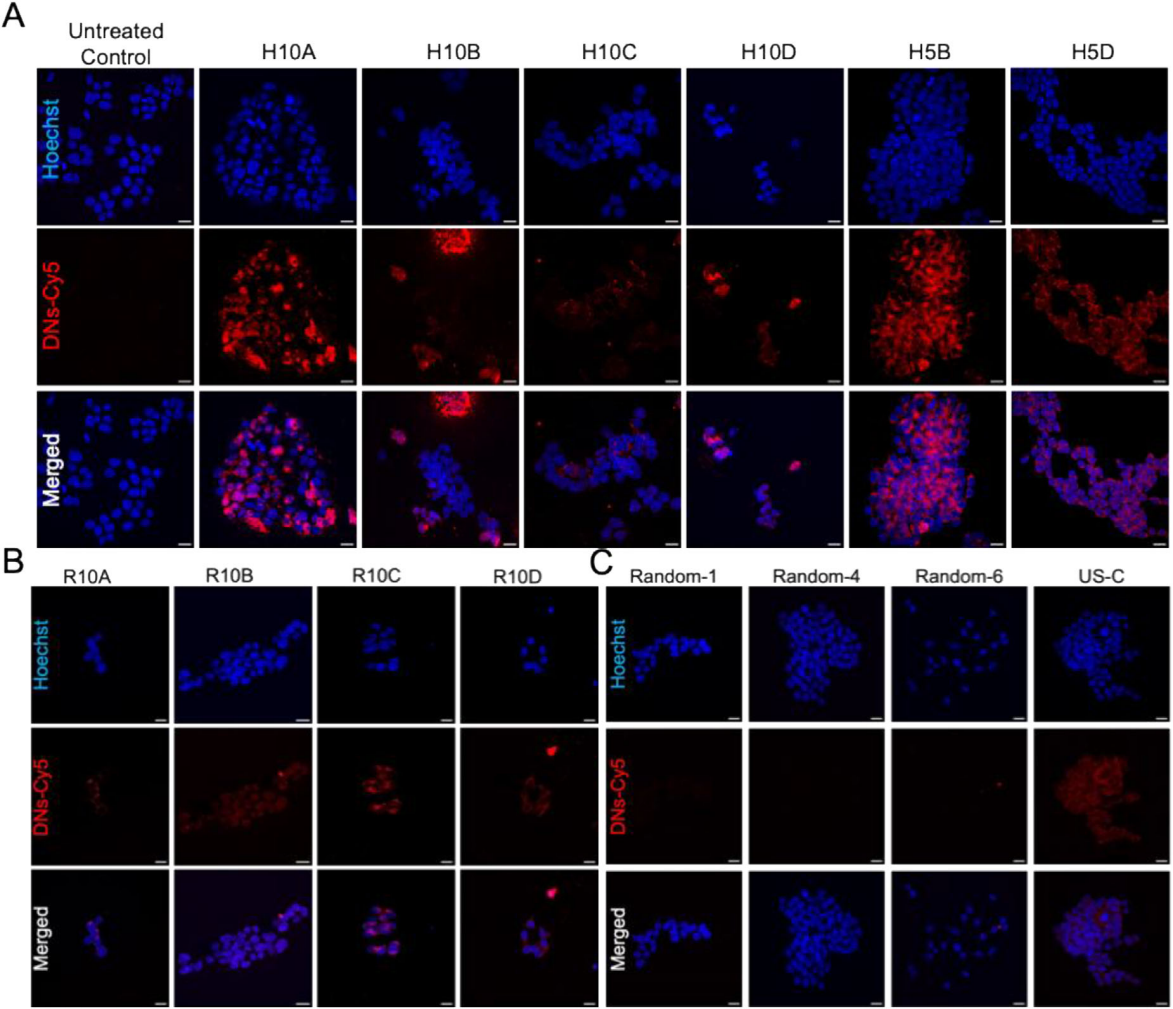
Qualitative assessment of cellular uptake of final candidates in HEK293T using laser scanning confocal microscopy. (A) Confocal images of cells treated with HEK293T‐selected DNA nanostructure (DNs‐Cy5). (B) Confocal images of cells treated with RAW cell‐selected DNA nanostructure. (C) Confocal images of cells treated with randomly selected DNA nanostructure. For all panels, the red channel represents DNs‐Cy5 uptake, and the bottom panels represent merged images of all channels with nuclei stained with Hoechst dye (blue). Scale bar: 20 µm.

In comparison, RAW 264.7 cells demonstrated more uniform internalization; cells within a single focal plane presented comparable signal intensities. There was no obvious nuclear‐associated fluorescence observed for any of the tested DNA nanostructures. While some candidates displayed punctate fluorescence patterns, others exhibited a more diffuse intracellular distribution, highlighting differences in their cellular localization profiles (Figure S24).

In A549 cells, overall fluorescence intensity was visibly reduced compared to other cell lines under identical acquisition parameters. The signal intensity was visibly lower in H5B, H5D, and H10D among candidates selected from HEK293T. Furthermore, candidates selected from RAW264.7 exhibited weaker uptake compared to the HEK‐selected group. Interestingly, while the majority of random controls showed negligible internalization, the US‐C displayed significantly higher fluorescence intensity than any other candidate in A549 cells. This was similar to what we observe in the flow cytometry data, where US‐C showed the highest number of Cy5‐positive cells compared to the rest of the candidates (Figure S25).

These observations reveal cell‐type‐dependent differences in internalization efficiency and subcellular distribution of the DNA nanostructure candidates. The pronounced heterogeneity and occasional nuclear entry in HEK293T cells contrast with the more homogeneous, cytosol‐restricted uptake in RAW 264.7 cells. The low fluorescence in A549 cells may reflect reduced uptake capacity of the constructs in the nonselected cell line. The elevated signal from the US‐C underscores the limitation of the method that some nonselected candidates that were lost from the selection pool might have some interesting behavior. The selection workflow favors structures that balance multiple properties, including cellular uptake, structural stability, nuclease resistance, and amplification efficiency across iterative rounds. Consistent with this, stability testing of the finalized candidates revealed that the enriched structures generally exhibited greater stability in biological media and serum‐free conditions over the 4 h incubation period used during selection compared to non‐enriched and random controls (Figure S26).

In contrast, although US‐C displayed strong cellular uptake, its stability decreased substantially at the 4 h time point relative to the enriched candidates. While US‐C remained largely intact at earlier time points, its reduced stability during prolonged incubation may have limited its persistence throughout the selection, recovery, and amplification workflow. This could explain why US‐C was not enriched during selection despite its favorable uptake properties.

To further assess whether the uptake properties of the selected DNA nanostructures could be generalized across different cellular contexts, we evaluated their internalization in three additional cell lines: MDA‐MB‐231, MCF‐7, and HeLa. In MDA‐MB‐231 cells, all candidates exhibited higher percentages of Cy5‐positive cells than in A549 cells, indicating generally enhanced uptake. Notably, three of the four non‐enriched candidates showed uptake in more than 90% of cells. Among the enriched candidates, only H5B and H5D achieved similarly high uptake, whereas the remaining enriched candidates displayed substantially lower uptake than non‐enriched candidates in MDA‐MB‐231 cells, highlighting the strong cell type‐dependence of nanostructure uptake (Figure 6). Confocal microscopy revealed a similar trend, with H5B and H5D displaying markedly brighter Cy5 fluorescence compared to the other enriched candidates, while Random‐4, Random‐6, and Control‐C also showed strong Cy5 fluorescence signals (Figure S27).

**FIGURE 6:**
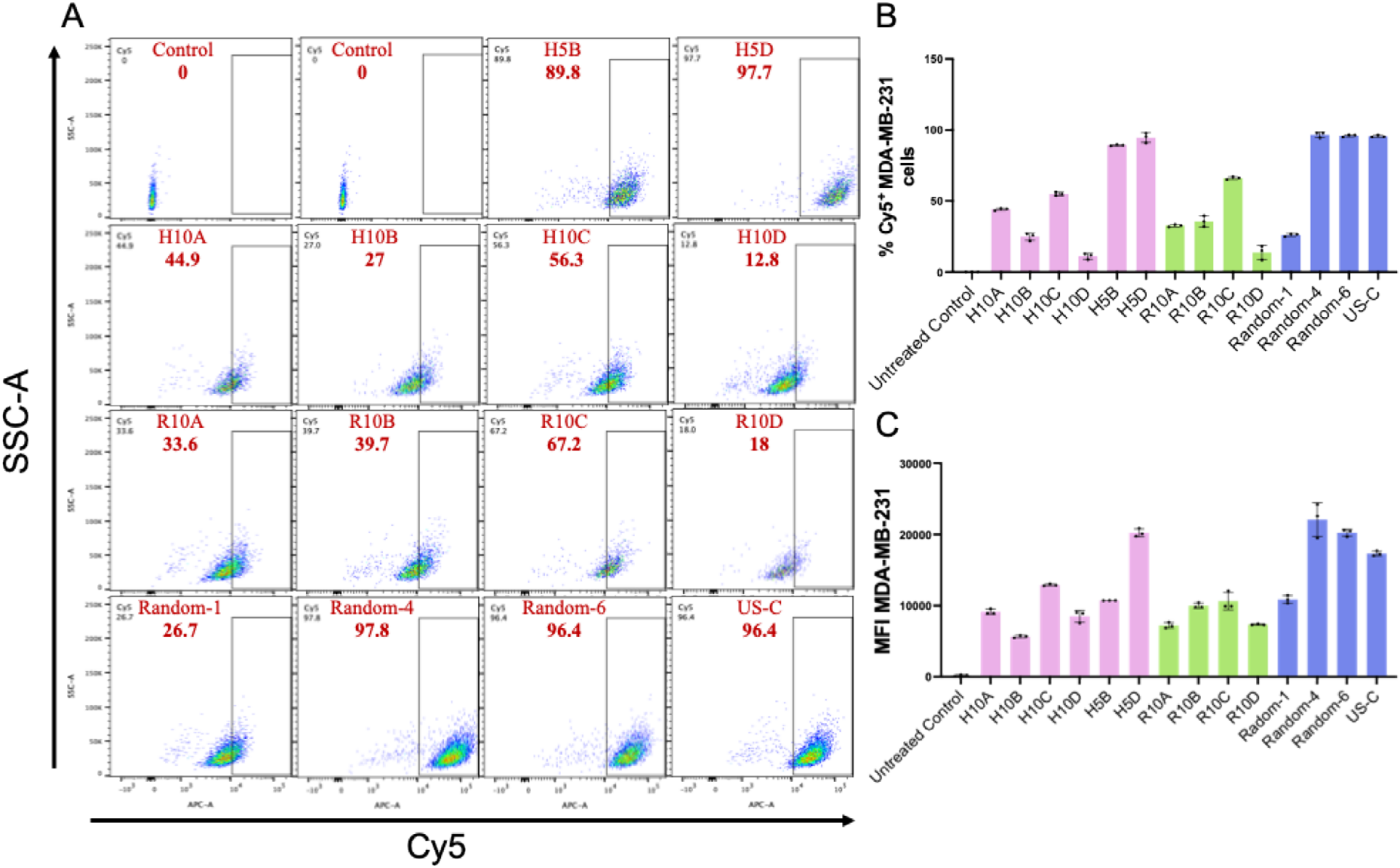
Quantification of DNA nanostructure internalization via flow cytometry in MDA‐MB‐231 cells. (A) Representative flow cytometry scatter plots of MDA‐MB‐231 cells treated with finalized DNA nanostructure candidates. (B) Quantification of Cy5+ cells calculated from scatter plots shown in (A). (C) Quantification of MFI of Cy5 under live cell population. For this experiment, 10 000 events were recorded per sample. Samples were acquired in triplicate (*n* = 3). The experiment was performed independently twice (*N* = 2) with similar results; representative data from one experiment are presented. Error bars indicate mean ± SD.

A similar pattern was observed in MCF‐7 (Figure S28) and HeLa cells (Figure S29), where non‐enriched candidates generally outperformed enriched candidates. In both cell lines, Random‐4, Random‐6, and unselected control‐C displayed the highest uptake, while H5B, H5D, and H10D were the best‐performing enriched candidates. In contrast, candidates enriched in RAW264.7 cells showed little or no uptake across these additional cell lines.

These findings suggest that the uptake properties of the candidates are highly cell type‐dependent and that structures enriched in one cellular context may not necessarily exhibit superior uptake across all cell lines.

### 2.6 Structure Selection in Cancer Cell Lines

The cell lines HEK293T and RAW264.7 are well known for their capacity to take up DNA making them useful model systems for developing and evaluating cellular selection strategies. To test if this approach also works on cells with more clinical relevance, we created a new DNA nanostructure library using the same set of 36 initial fragments. The library was generated following the same preparation pipeline as described in the previous section and included fragment annealing, ligation, Exo‐III digestion, amplification, λ‐exo digestion, and refolding (Section 2.1). The resulting library was subjected to iterative cellular selection in two widely used cancer cell lines: MDA‐MB‐231, a triple‐negative breast cancer cell line, and A549, a lung cancer cell line (Figure 7A). Each selection round followed the same incubation conditions established for HEK293T and RAW264.7 (2 µg library, 4 h incubation at 37°C), and internalized structures were recovered, amplified, and refolded between rounds using the workflow described in Section 2.2. We performed 5 rounds of selection using the same approach as before and sequenced the result of each round using ONT sequencing. In both cancer cell lines, we saw a gradual increase in reads mapping to the human genome (Figure 7B) and a general preference for shorter structure genomes that was slightly larger in A549 combined with the appearance of a small second peak at around 240 nt genome length (Figure 7C). The preference for fragment types was generally similar to the non‐cancer cell lines, that is, favoring linear fragments and disfavoring kissing‐loop‐containing fragments, but interestingly the fold changes after five rounds in cancer cell lines were stronger than after 10 rounds in HEK/RAW cells (Figure 7D). The tracking of UMI labels on DNA nanostructures also showed a similar magnitude of enhancement of individual structures as in the ONT data after 10 rounds in the non‐cancer cell lines (Figure 7E and Figure S7). This five‐round selection was performed primarily as a proof of concept to evaluate whether the workflow could be successfully transferred to cancer cell lines. The results demonstrate that the selection strategy is not limited to the original HEK293T and RAW264.7 model systems but can also be applied to clinically relevant cancer cell lines. Notably, substantial enrichment was observed after only five rounds of selection, suggesting that the selection pressure in these cancer cell lines may be stronger than in the original model systems. These findings indicate that cancer cell lines may serve not only as a more translationally relevant platform for cellular selection but also as a potentially more efficient one.

**FIGURE 7:**
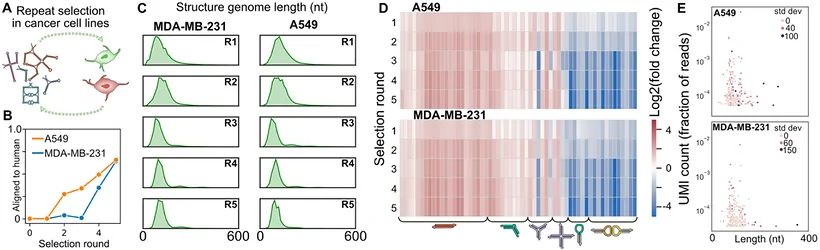
Cell uptake experiments in lung (A549) and breast (MDA‐MB‐231) cancer cell lines, sequenced on ONT. **(A)** Schematic showing the iterative selection of DNA library in two different cell lines. **(B)** Fraction of reads aligning to the human genome. **(C)** Length of structure genomes from the cell uptake experiments in both cell lines, after filtering out human genome contamination. **(D)** Change in relative abundance of structure fragments compared to the initial library in two cell lines, filtered to remove reads mapping to the human genome. **(E)** Distribution of abundance, genome size (excluding primer sites), and length variability of UMI‐labeled structure genomes after 5 rounds of selection in two cancer cell lines.

## 3 Discussion

Developing structures that can specifically target and deliver drugs to certain cell types is a major goal of DNA nanotechnology. The strength of DNA nanotechnology is the ability to precisely control size, shape, and mechanical properties to optimally achieve a target function. Although several recent studies have demonstrated that DNA nanostructure design choices greatly influence uptake in different cell types [7, 9, 34, 35], the optimal combination is unknown, and current methods are limited to testing structures one by one.

Here we attempt to invert the process by using cell uptake as a selection pressure to discover structures that are preferentially taken up by cells from a large initial library. In order for selection to function, the DNA structures must be compatible with amplification and sequencing; this is not possible with DNA origami and tile techniques where the composite strands disassemble when heated. Instead, we base our approach on DNA nanostructures that fold from one long single strand that we achieve by randomly ligating smaller DNA nanostructure fragments assembled from 1‐5 oligonucleotides. Although this limits the number of possible structural motifs, DNA nanostructures that fold from a single long strand are easier to scale up than other approaches as the single genome can be amplified enzymatically. When analyzing our assembled structure genomes after selection, we realized that many structures did not contain the expected ordering of fragment sequences after ligation. This could be due to several reasons in assembly: poor assembly of certain constituent fragments, incorrect palindromic ligation, or PCR artifacts. These parameters could be optimized to drive the assembly of expected combinations. Future library designs could reduce these events through the implementation of more stringent ligation interfaces, such as longer or computationally designed recognition sequences with reduced cross‐reactivity, or through the adoption of hierarchical assembly strategies that enforce stricter control over fragment order and connectivity. These approaches would reduce the proportion of off‐target ligation products and improve the structural integrity of the library. Also, the integration of computational tools for predicting DNA nanostructure assembly, thermodynamic stability, and structure–function relationships could significantly enhance library design. Combining experimental selection with predictive modeling frameworks may reduce unproductive library diversity, improve assembly fidelity, and increase the overall efficiency of future screening campaigns.

Intriguingly, secondary and 3D structure predictions still indicate that we achieved complex branched structures with diverse shapes (Figure S19).

The underlying assumption that cells preferentially uptake DNA nanostructures with certain shapes and mechanics appears to vary highly among cell types. In this first proof‐of‐concept work, we initially chose to test cell lines that are known to abundantly take up DNA: a macrophage‐like cell line (RAW264.7) and a cell line well known for its ease of transfection (HEK293T). Using these cells, we demonstrated that DNA nanostructures could be amplified, sequenced, and refolded for repeat selection from cell lysate. However, the macrophage‐like cells appeared very promiscuous in their uptake preferences making them an important control but not ideal for discovering cell‐specific targeting. This was true to a lesser extent in the HEK293T cells, where we were able to discover and select for DNA nanostructures that were taken up more effectively than randomly selected DNA nanostructures. Intriguingly, when we tested our monoclonal DNA nanostructure candidates for uptake in a cell line not used in the selection: the human lung cancer cell line A549, it showed a great variety of uptake preferences of fluorescently labeled structures, indicating that cancer cell lines may be a good target for structure type selection. We later tested our selection approach in two commonly used cancer cell lines, MDA‐MB‐231 and A549. Interestingly, we see similar trends in cellular uptake preference, but at a higher magnitude after only five rounds of selection, indicating that they create a stronger uptake selection pressure than transfection/macrophage cell lines and could be a more suitable model for selection going forward. In several of the cellular selection experiments, we saw a gradual increase of structure genomes that appeared to be chimeras between our structure fragments and genomic DNA; we hypothesize that these are created if partially degraded DNA structures act as primers in the post‐lysis PCR. This could be reduced by biotinylating structures before cell incubation allowing for the removal of genomic DNA before PCR amplification.

In any synthetic selection technique, it is important to be mindful that results may be a combination of several parallel selection pressures. Although we primarily discuss cell type‐specific uptake here, the selection process we see is likely to also be influenced by the stability of the DNA structures in the cell culture: structures that degrade are less likely to be amplified and sequenced, something that is a positive property of a DNA nanostructure for drug delivery. Stability testing in cell culture media of selected and nonselected structure candidates did indeed reveal differences in structural stability. This could be explored further by parallel selection experiments where the incubation time is varied. PCR bias, where some structures are more preferentially amplified, is a more concerning selection pressure, as it could create the illusion of cell selection. Here we have performed control experiments where we only amplify the structures without cellular incubation and not seen any clear trends, but this is still an important factor to keep in mind. In our results, we see a structure unselected control‐C, that is, not enhanced for selection in either cell line but still appears to be effectively taken up; this may be due to the abovementioned factors (poor stability or amplification) or may just be a random effect when the selection pressure is not strong.

Our sequencing results after 10 rounds still contained a diverse set of DNA nanostructures that have been enhanced by a mixture of selection pressures. It is only feasible to individually test a handful of these structures and knowing what candidates to pick is not trivial, and current bioinformatics tools are poorly adapted to this type of data. Future computational work may be able to deconvolute the parallel selection pressures, for example, by comparing multiple selection experiments or using structure prediction at scale to group structures with similar features. This may allow us to find better candidates, either in new experiments or in the sequencing data we publish here.

In this work, we only use a library of DNA nanostructures varied in size, shape, and mechanics to understand the effect on cellular uptake preferences. It is known that the addition of targeting groups greatly influences the cell type‐specific targeting of structures [4, 36]. In future work, DNA aptamers could be directly integrated in DNA loops and used to target surface groups on target cells increasing the selection pressure. Aptamers integrated in the structure genome could be directly amplified for repeat selection and sequencing. Other functional groups such as proteins or peptides cannot be directly integrated in the genome but could be conjugated to DNA handles complementary with loops in the DNA structures and be added to the library in each selection round.

Our results demonstrate that the preference of DNA nanostructure shape is cell‐type dependent. The predicted secondary structure of selection‐enriched structures has more loops compared to randomly chosen structures that display longer stems and fewer branches. HEK293T cells appear to preferentially internalize flexible, loop‐rich architectures, while A549 cells favor thermodynamically stable, rigid structures with compact, linear morphologies. In contrast, RAW264.7 macrophages have consistently high uptake across both enriched and non‐enriched nanostructures, consistent with their phagocytic phenotype and reduced sensitivity to fine structural parameters.

The structures selected for in this study could either stick to the cell surface or be taken up inside the cells by several mechanisms. It is feasible to use the tools developed here to study the uptake mechanisms in more detail, for example, by chemically blocking certain uptake routes [37, 38, 39] or conducting selection at 4°C could reduce active uptake and help distinguish uptake‐dependent from surface‐binding species. Alternatively, we could use cell fractionation in each round to select for structures that end up in certain cellular compartments, or DNase treatment prior to lysis to remove surface‐bound structures, while varying incubation times could shift the selection pressure toward either rapid internalization or long‐term intracellular persistence. It is conceivable that this type of selection could even be used in vivo where a library is injected into an animal followed by selection from certain tissue types or tumors. The structures designed here are highly modular and could be readily functionalized with a variety of therapeutic or imaging cargos using established DNA conjugation and hybridization strategies. They can be directly loaded with intercalating anticancer drugs such as doxorubicin [40]; complementary overhangs could be added to integrate nucleic acid therapeutics such as antisense oligonucleotides (ASO) or small interfering RNAs (siRNA), acting as delivery vehicles for gene silencing. This would further strengthen the translational relevance of the platform.

In conclusion, we have developed a method for turning DNA nanostructure cell targeting from a design to a selection process. We have developed a new type of DNA nanostructure compatible with amplification and sequencing and developed the processes needed to use cellular uptake as a selection pressure. We have demonstrated that there is a cellular preference for certain types of DNA nanostructures, but the specificity is greatly dependent on the cell type. We hope that this can be a tool to discover DNA nanostructures that would be intractable to find with current methods to potentially target diseases lacking efficient therapies today.

## 4 Experimental Section

### 4.1 DNA Library Preparation

We employed a library of orthogonal 20‐nucleotide sequences as the foundation for designing DNA fragments with diverse geometries and sizes. These sequences were optimized to have similar melting temperatures, balanced GC content, low cross‐interaction, and no homopolymer repeats. From these, we created fragments including linear duplexes of varying lengths (10–20 nt), bulged fragments (20 nt) with bulge sizes ranging from 1 to 5 nt, hairpin structures (10–18 nt), kissing loops (10–20 nt), a three‐way junction, a four‐way junction, and a four‐way junction containing a thymine (T) at the junction. We also created an amplification loop hairpin with two adjacent primer binding sites flanked by a 10 nt random sequence region that functions as UMIs. The design and structural modelling of these fragments were performed using oxView [41] and oxDNA [32], computational tools for DNA nanostructure simulation. DNA oligonucleotides were purchased from IDT at a stock concentration of 100 µM and diluted to 10 µM in nuclease‐free water for further use. Complementary strands for each DNA fragment were annealed in 1 × T4 DNA ligase buffer (NEB) using a temperature ramp from 90°C to 4°C over 30 min. For each fragment, 45 µL of the 10 µM oligonucleotide mixture was combined with 5 µL of 10 × DNA ligase buffer (NEB), yielding an effective concentration of 9 µM per fragment. All 37 annealed fragments were then pooled together in equimolar amounts and mixed with T4 DNA ligase (NEB, 2000 U/µL) to generate the ligated library. This was incubated for 10 h at 16°C, followed by an overnight hold at 4°C.

**TABLE 2:** PCR reaction setup of exonuclease digested DNA library.

Components	Volume (µL)
2 × DreamTaq master mix	25
Fwd primer (10 µM)	2
Bwd primer (10 µM)	2
Lib+ExoIII	2
H2O	19
Total vol. (µL)	50

**TABLE 3:** End‐Prep reaction setup of DNA library for nanopore sequencing.

Components	Volume (µL)
DNA amplicon	24.5
DCS	0.5
Ultra II end‐prep reaction buffer	3.5
Ultra II end‐prep enzyme mix	1.5
**Total**	**30.0**

**TABLE 4:** Adaptor ligation reaction setup of DNA library for nanopore sequencing.

Components	Volume (µL)
End‐prepped DNA	30.0
Ligation buffer (LNB)	12.5
NEB next quick T4 DNA ligase	5.0
Ligation adapter (LA)	2.5
**Total**	**50.0**

**TABLE 5:** Flow cell loading reaction setup for nanopore sequencing.

Components	Volume (µL)
Sequencing buffer (SB)	15
Library beads (LIB)	10
DNA library	5
**Total**	**30**

**TABLE 6:** PCR reaction for amplification of selected candidates using purified lysate as a template.

Components	Volume (µL)
2 × DreamTaq master mix	50
Fwd primer (10 µM)	2.5
Bwd primer (10 µM)	2.5
Template (purified lysate)	5
H_2_O	40
Total vol. (µL)	100

**TABLE 7:** PCR amplification reaction of selected candidates using commercial DNA fragment (synthesized from ANSA) as a template.

Components	Volume (µL)
2 × DreamTaq master mix	50
Fwd primer (10 µM)	1
Bwd primer (10 µM)	1
Template (purified lysate)	0.5
H_2_O	47.5
Total Vol (µL)	100

#### 4.1.1 Exonuclease Treatment and PCR Amplification

Following ligation, the library was treated with Exonuclease III (Exo III; NEB, 100 U/µL), which selectively digests dsDNA from 3′ to 5′ at linear or nicked sites. Because Exo III progressively resects dsDNA—producing intermediates with short single‐stranded extensions and ultimately ssDNA—this step preferentially removes unligated or partially folded fragments while preserving stable, fully assembled structures. Exonuclease digestion was performed using the following temperature program: 37°C for 30 min, followed by 70°C for 20 min to heat‐inactivate the enzyme. A negative control lacking enzyme was processed in parallel. Reactions were set up as shown in Table 1:

The Exonuclease III–digested library served as the template for PCR amplification using primers specific to the UMI‐containing amplification loop. In principle, only structures that had successfully incorporated the amplification loop should be amplified under these conditions. To optimize amplification while minimizing nonspecific products, we tested annealing temperatures ranging from 50°C to 60°C and used primers at a final concentration of 0.4 µM. A template concentration of 1 ng/µL was used, and multiple cycle numbers (10, 15, 20, and 25 cycles) were evaluated to avoid overamplification, nonspecific products, or primer depletion. The PCR reaction setup for a 50 µL reaction volume is shown in Table 2.

Based on these optimization experiments, 60°C was selected as the annealing temperature and 15 cycles as the optimal amplification regime. PCR reactions were prepared using DreamTaq 2 × Master Mix (Thermo Fisher Scientific) in 50 µL reaction volumes. The thermal cycling protocol consisted of an initial denaturation at 95°C for 2 min, followed by 15 cycles of 95°C for 30 s, 60°C for 30 s, and 72°C for 30 s, with a final extension at 72°C for 10 min. Following amplification, products were purified using AMPure beads to remove residual primers and small contaminants.

#### 4.1.2 AMPure Purification Protocol

PCR amplicons were purified using solid‐phase reversible immobilization (SPRI) with AMPure XP paramagnetic beads (Beckman Coulter). Beads were added to each sample at a 1.8 × bead‐to‐sample volume ratio (324 µL of thoroughly resuspended beads to 180 µL of PCR product) and mixed on a rotary mixer for 5 min at room temperature to allow DNA binding. Samples were placed on a magnetic stand, and the supernatant was discarded once bead separation was complete. The bead‐bound DNA was washed twice with 200 µL of freshly prepared 75% ethanol. Beads were briefly air‐dried to remove residual ethanol while avoiding over‐drying. Purified DNA was then eluted in 100 µL of nuclease‐free water and subsequently processed either for nanopore sequencing or for lambda exonuclease digestion and refolding prior to cell selection assays.

#### 4.1.3 Lambda Exonuclease Digestion and Refolding

To prepare the library for subsequent rounds of cell selection assays, the purified dsDNA library was subjected to lambda exonuclease digestion to convert the library into ssDNA. Lambda exonuclease (NEB; 5 U/µL) is a 5′→3′ exonuclease that specifically binds to 5′‐phosphorylated dsDNA. Upon binding, the enzyme selectively digests only the phosphorylated strand, progressing unidirectionally from the 5′ end. As nucleotides are removed, the complementary strand remains intact, generating long ssDNA that facilitates downstream refolding behavior. Following the manufacturer's recommendation of 1 unit of enzyme per ∼1 µg of purified dsDNA, the digestion reactions were assembled based on Qubit‐quantified dsDNA concentrations. The reaction was incubated at 37^°^C for 30 min, followed by heat inactivation at 75^°^C for 10 min to terminate enzymatic activity. Post‐digestion Qubit measurements confirmed that most of the library had been converted to ssDNA: the dsDNA signal was substantially reduced, whereas the ssDNA concentration increased, opposite to the predigestion profile. This shift is consistent with efficient strand‐specific degradation by lambda exonuclease.

The digested library was then refolded in 1 × T4 DNA ligase buffer (NEB) using a controlled temperature ramp to promote recovery of the nanostructures' intended secondary conformations.

### 4.2 AFM Sample Preparation and Imaging

Samples were diluted 5‐fold in AFM buffer (1 × TE buffer (Thermo Fisher Scientific) supplemented with 10 mM MgCl_2_ (Thermo Fisher Scientific)). Ten microliters of diluted sample solution were applied to freshly cleaved mica and allowed to incubate for 30 s. Four microliters of 5 mM NiSO_4_ were added to the mica on top of the sample and allowed to incubate for another 4 min 30 s at room temperature. The mica was then washed by applying and removing 1 mL of AFM buffer using a pipette. After washing, 1.5 mL of AFM buffer was added to the mica for imaging. AFM imaging was performed in liquid using a JPK Instruments Nanowizard 3 Ultra with a Si_3_N_4_ Olympus Biolever Mini cantilever in a Bruker Hyperdrive glass block. Images were taken in AC Mode with a frequency in the range 25–35 kHz and a setpoint of 70%–80%.

### 4.3 Cellular Selection

We conducted 10 rounds of cellular selection experiments using two cell lines, HEK293T (RRID:CVCL_0063) and RAW264.7 (RRID:CVCL_0493), and five rounds of selection in A549 (RRID:CVCL_0023) and MDA‐MB‐231 (RRID:CVCL_0062). Additional cell lines, including MCF‐7 (RRID:CVCL_0031) and HeLa (RRID:CVCL_0030) were solely used for candidate validation. All cell lines were cultured in Dulbecco's Modified Eagle Medium (DMEM) supplemented with 10% fetal bovine serum (FBS) and 1% penicillin‐streptomycin. Cells were maintained at 37°C in a humidified incubator with 5% CO_2_. For subculturing, HEK293T, A549, and MDA‐MB‐231 cells were detached using trypsinization and reseeded following standard protocols. In contrast, RAW264.7 cells, being adherent macrophages, were harvested using a cell scraper and subsequently subcultured into fresh medium. All cell lines were regularly tested and confirmed free of mycoplasma contamination.

#### 4.3.1 DNA Library Treatment and Uptake Protocol

Cells were seeded in six‐well plates one day prior to the experiment to ensure adequate adherence and optimal confluency on the day of the experiment. Before treatment, the culture medium was removed and the cells were washed once with serum‐free DMEM. Next, we added 2 µg of the refolded DNA library sample to each well in serum‐free DMEM to facilitate cellular uptake without serum interference. Cells were incubated with the DNA library for 4 h at 37°C and 5% CO_2_ to allow cellular uptake.

#### 4.3.2 Post‐Incubation Processing

After incubation, the treatment medium was removed, and cells were washed three times with ice‐cold 1 × PBS to remove any loosely associated surface‐bound DNA structures. For HEK293T cells, washing was performed gently to avoid dislodging the adherent cells, as excessive washing could reduce the cell population available for downstream analysis. Cells were lysed in 200 µL of RIPA lysis buffer supplemented with Proteinase K (final concentration 500 µg/mL) and incubated on ice at 4°C for 5 min to ensure efficient cell lysis. After lysis, the lysates were collected into 1.5 mL microcentrifuge tubes, and an additional 200 µL of 1 × PBS was added to rinse well and recover all possible cellular contents. The collected cell lysates were incubated at 55°C for 30 min with intermittent vortexing to facilitate complete cellular digestion and release of intracellular contents. After this, the samples were centrifuged at 4,000 rpm for 10 min at room temperature to separate the supernatant from any debris. This preparation ensured that the maximum amount of internalized DNA nanostructures could be retrieved for further analysis and selection processes. The supernatant was then transferred to a separate tube for purification of internalized structures.

#### 4.3.3 Cell Lysate Processing

Cell lysate DNA was purified using AMPure XP paramagnetic beads (Beckman Coulter). The 700 µL sample was split into two tubes (350 µL per tube), and AMPure XP beads were added at a 1.8 × ratio (630 µL per tube). Samples were mixed thoroughly and incubated for 10 min at room temperature to allow DNA binding. Following magnetic separation, the supernatant was removed, and the bead‐bound DNA was washed twice with 500 µL of freshly prepared 75% ethanol. Beads were air‐dried briefly, and DNA was eluted in nuclease‐free water with a 10 min incubation before magnetic separation and transfer of the eluate to a fresh tube for further amplification and downstream processing.

#### 4.3.4 PCR Amplification of Lysate

PCR reactions were set up using the conditions described in Section 4.1.1. Amplification was performed using DreamTaq 2 × Master Mix (Thermo Fisher Scientific) in a total reaction volume of 1 mL, which was subsequently divided into ten 100 µL reactions, with template DNA at a final concentration of 1 ng/µL. Thermal cycling consisted of an initial denaturation at 95°C for 2 min, followed by 15 cycles of denaturation at 95°C for 30 s, annealing at 60°C for 30 s, and extension at 72°C for 30 s, with a final extension at 72°C for 10 min.

The amplified products were purified using AMPure XP beads following the lysate purification protocol. A portion of the purified amplicons was used for nanopore sequencing, while the remaining sample was subjected to lambda exonuclease digestion as described in Section 4.1.3 and subsequently refolded in 1 × T4 ligase buffer.

### 4.4 Nanopore Sequencing

Amplicon libraries of HEK293T and RAW264.7 were prepared for Nanopore sequencing on the MinION platform with Flongle flow cells using the ONT Ligation Sequencing Kit (SQK‐LSK114), following the manufacturer's recommended workflow for the *Ligation Sequencing Amplicons V14* protocol.

#### 4.4.1 End Prep for Nanopore Sequencing

End Prep was performed to prepare the DNA ends for adapter ligation. Purified PCR amplicons were diluted in nuclease‐free water to achieve 100 fmol input, assuming an average fragment length of 0.5 kb (32.5 ng DNA). DNA concentration was determined by Qubit, and the input volume was adjusted accordingly. End‐prep reactions (30 µL total volume) were assembled as shown in Table 3:

Reactions were mixed gently and incubated at 20°C for 5 min followed by 65°C for 5 min. DNA was purified using AMPure XP beads (30 µL), incubated for 5 min at room temperature, and magnetically separated. Beads were washed twice with freshly prepared 80% ethanol, briefly air‐dried, and DNA was eluted in 30 µL nuclease‐free water.

#### 4.4.2 Adapter Ligation

Adapter ligation was performed to attach sequencing adapters to the DNA ends. To retain all DNA fragments, short fragment buffer (SFB) was used during post‐ligation cleanup. Ligation reactions (50 µL total volume) were assembled as in Table 4:

The reaction was incubated for 10 min at room temperature. Post‐ligation, the sample was purified using AMPure XP beads (20 µL beads in 50 µL of reaction vol.) incubated on a rotary mixer for 5 min. The samples were briefly centrifuged and placed on a magnetic rack to remove supernatant, and the pelleted beads were washed twice with SFB, briefly air‐dried, and eluted in 7 µL Elution Buffer. Library concentration was quantified using a Qubit fluorometer, and the final library was diluted to 5–10 fmol prior to sequencing.

#### 4.4.3 Flow Cell Check and Sequencing

Flongle flow cells were inspected for active pores using MinKNOW software prior to sequencing. The DNA library was prepared at 5–10 fmol in 5 µL of elution buffer, corresponding to 3.25 ng of DNA for 10 fmol assuming an average amplicon length of 0.5 kb. Flush solution was prepared using 117 µL flow cell flush (FCF) and 3 µL flow cell tether (FCT) and used for priming the flow cell. The library sample was prepared as in Table 5:

The prepared library was loaded carefully to avoid air bubbles onto the flow cell, and sequencing was performed using super‐accurate basecalling with a minimum read length threshold of 20 bp and POD5 output format.

### 4.5 Nanopore Sequencing and Native Barcoding

The amplicon libraries obtained from the MDA‐MB‐231 and A549 selection experiments were sequenced using the Native Barcoding Kit 24 V14 (SQK‐NBD114.24, Oxford Nanopore Technologies) on a MinION platform. The workflow was largely identical to the standard ligation sequencing protocol described above, with the addition of a native barcoding step to enable multiplexing of samples. The PCR amplicons from rounds R1 to R5 were purified using AMPure XP beads and quantified using the Qubit dsDNA high sensitivity assay. DNA concentrations were converted to molar concentrations assuming an average amplicon length of 500 bp, and 200 fmol of each sample (64.9 ng of DNA) was used as input for library preparation.

Unlike the standard ligation sequencing workflow, each sample was processed individually during the end‐repair and dA‐tailing step. Following end preparation, samples were purified using AMPure XP beads and ligated to unique native barcodes according to the manufacturer's instructions. Care was taken to assign a distinct barcode to each sample to ensure accurate identification during downstream demultiplexing. After barcode ligation, all samples were pooled in equimolar amounts and subjected to adapter ligation following the standard ligation sequencing workflow. The adapter‐ligated library was purified using AMPure XP beads, and the final library concentration was determined using the Qubit dsDNA high sensitivity assay. A total of 100 fmol of the pooled library (32.45 ng of DNA) was loaded for sequencing.

Prior to library loading, flow cell quality was assessed using MinKNOW to verify the availability of sufficient active nanopores. The sequencing library was prepared by combining the pooled DNA library with sequencing buffer and library beads supplied by Oxford Nanopore Technologies and loaded onto an R10.4.1 MinION flow cell according to the manufacturer's priming and loading protocol. Sequencing runs were performed using MinKNOW with super‐accurate basecalling enabled, and raw data were exported in POD5 format. Reads were subsequently basecalled and demultiplexed according to their native barcode assignments using Oxford Nanopore Technologies software.

### 4.6 Illumina Sequencing

We tested a total of five samples for illumina sequencing, which were: the initial library, HEK293T‐R5, HEK293T‐R10, RAW264.7‐R5, and RAW264.7‐R10.

#### 4.6.1 Illumina Library Preparation

Libraries were prepared using a two‐step PCR strategy to append illumina‐compatible adapters and unique dual indices.

##### 4.6.1.1 Primary Amplification (PCR‐1)

The initial PCR was performed in a 25 µL reaction volume using 12.5 µL 2 × KAPA HotStart ReadyMix (Roche), 1 µM each of custom primers containing Illumina‐specific overhangs and library‐specific regions, and 5–10 ng of template DNA. Cycling conditions consisted of an initial denaturation at 95°C for 3 min, followed by eight cycles of 98°C for 20 s, 60°C for 15 s, and 72°C for 1 min, with a final extension at 72°C for 1 min.

Primers used for this reaction were:

FWD Primer: 5′‐

ACACTCTTTCCCTACACGACGCTCTTCCGATCTCCAGCACGTGATATGTATATGT‐ 3′

BWD Primer: 5′‐

GTGACTGGAGTTCAGACGTGTGCTCTTCCGATCTCCTACACGCGTACATGTATAGA‐ 3′

##### 4.6.1.2 Indexing and Adapter Ligation (PCR‐2)

Secondary PCR amplification was used to incorporate P5/P7 adapters and unique indices. The 40 µL reaction comprised 20 µL 2 × KAPA HiFi HotStart ReadyMix, 10 µL of purified PCR‐1 product, and 1 µM indexed primers. The profile included an initial denaturation at 98°C for 2 min, followed by eight cycles of 98°C for 20 s, 55°C for 30 s, and 72°C for 30 s, concluding with a 2 min extension at 72°C. Following each PCR step, products were purified using AMPure XP beads (Beckman Coulter) at a 1.8:1 volumetric ratio. Purification efficiency and the removal of primer dimers were confirmed via 2% agarose gel electrophoresis.

#### 4.6.2 Quantification and Equimolar Pooling

Library concentrations were determined via relative qPCR using SYBR Green chemistry, with the “initial library” sample serving as the reference standard. Primer efficiencies were derived from Ct values and maintained within an acceptable range of 90%–110%. Based on these calculations, all the libraries were pooled in equimolar ratios to achieve a final concentration of ≈15 nM in a total volume of 54 µL.

### 4.7 Bioinformatics

#### 4.7.1 Sequencing Data Preprocessing for Downstream Analysis

Nanopore sequencing was performed on the MinION Mk1B device using the MinKNOW software. Sequencing reads were pre‐filtered, setting the minimal Q score to 10. Basecalling was performed using Dorado's super‐accurate basecalling. The fastQ files containing the passed and basecalled reads were concatenated for downstream processing.

The Illumina reads were split by index into five groups representing the five sequenced libraries. Illumina sequencing results in two paired‐end reads up to 300 nt long each, read from either end. Before further processing, these paired reads were fused using the tool Pear [42] into one single nonoverlapping read.

#### 4.7.2 cONcat: Mapping of the Structure Genomes to Initial Library Fragments

To identify and decode the known fragments in each read, that is, the structure genome, the concatenated fastQ files were run through cONcat, a software developed by Petri et al. [29]. Here, the concatenated FASTQ files were given to the software together with the sequences of the fragments making up the components of the initial library (Table S1). The fragment sequences were mapped onto the reads using an edit‐distance‐based recursive covering algorithm with an identity threshold of 0.75. If present, the software returned a set of best‐matched fragments above the identity threshold for each read, including the start and end positions of the fragments within the reads, the fragment name, and edit distance of the mapping. This information was used to further decode general trends of fragment pairings, order, and frequency in the structure genomes throughout the selection process.

#### 4.7.3 UMI Extraction and Counting of Barcodes

To further analyze reads from the sequencing data and identify and count the prevalence of unique structure genomes, each read was characterized and sorted by its barcode, consisting of UMI1 and UMI2. To extract the UMIs from the sequencing data, concatenated FASTQ files were processed using Python, utilizing Bio.Align and Bio.SeqIO packages. To ensure that sequencing reads corresponded to true structure genomes, reads with a length below 80 nucleotides were discarded. The remaining reads were scanned for inclusion of the universal primer binding sites. Using the known primer sequences for the forward and reverse primers, primer binding sites were localized via pairwise local alignment within the first and last 90 nucleotides. Any read with a primer alignment score below the threshold was discarded. Since the ONT ligation sequencing kit (SQK‐LSK114) results in reads with mixed read directions, backward reads were turned into their reverse complement strand, resulting in a pool of “pure” forward reads. Via the forward primer alignment, UMI1 was defined to be the 10 nucleotides following the forward primer binding site. Similarly, UMI2 was defined as the 10 nucleotides prior to the backward primer alignment. UMI1 and UMI2 sequences were fused into a barcode, with each unique barcode denoting a unique structure genome. Reads were then sorted by barcode, and each barcode was counted to further analyze the enrichment of unique structure genomes.

#### 4.7.4 Detection and Filtering of Biological Sequences in Data

In order to detect instances of structure genomes that appear to be chimeras with genetic DNA from the cells, we aligned all reads to the GRCh38 [43, 44] human reference genome using minimap2 [27]. Reads that were mapped to the reference genome were filtered out and not used in during the selection process of candidates from Round 10 from HEK293T and RAW264.7 as well as control candidates. To control cell‐specific contamination, RAW264.7‐originating sequencing reads were also subjected to filtering against the mouse genome GRCm39 via Minimap2.

#### 4.7.5 Secondary Structure Prediction of Selected Candidates

##### 4.7.5.1 mFold

The secondary structures of selected DNA candidates were predicted using the Zuker mFold web server utilizing UNAfold software package configured for DNA folding [30]. The folding simulations were conducted under the following conditions: 100 mM [Na^+^], 10 mM [Mg^2^^+^], and a temperature of 37°C. The folding algorithm was configured with a percent suboptimality of 5%, ensuring that only structures within 5% of the minimum free energy (MFE) were considered. The upper bound on the number of computed foldings was restricted to 5 to prioritize the most thermodynamically probable architectures. A window parameter of 10 was applied to control the diversity of the generated structures, preventing the output of highly similar, redundant conformations.

The minimum Gibbs free energy (Δ*G*) of self‐hybridization was calculated for each sequence to quantify the energetic favorability of the predicted folds. To facilitate a normalized comparison across candidates of varying lengths, the stability density was calculated as follows:

$$\mathrm{Stability}\;\mathrm{Density}=\frac{\Delta G}{\mathrm{Sequence}\;\mathrm{Length}\;(\mathrm{nt})}$$

##### 4.7.5.2 oxDNA

NUPACK predicted secondary structure (DNA model, 37°C, 1 µM strand concentration, 1.0 M Na^+^) was converted into 3D starting configurations for oxDNA simulation. The initial configurations were constructed to preserve realistic backbone and base‐pairing distances while avoiding steric overlaps and overstretched bonds. The result was a configuration incorporating idealized B‐form helical stems (twist ≈ 34.3°/bp), 0.72 su adjacent nucleotide distance and 1.2 su base‐pair separation (1 su ≈ 0.85 nm). Structures were relaxed in two stages: an initial Monte Carlo (CPU‐based) simulation at 30°C and 1 M [Na^+^] (interaction type DNA) for 100 000 steps to remove any residual overlaps from the initial configuration. The second stage was a molecular dynamics simulation (GPU‐based) at 20°C, 1 M [Na^+^] (interaction type DNA) for 1 000 000 steps, dt = 0.003. The simulation was considered converged when the final potential energy per nucleotide was between –1.0 and –1.5.

### 4.8 Candidate Synthesis and Verification

#### 4.8.1 Amplification From Purified Lysate

The initial candidate series (H5A–H5D) was amplified using purified lysate as the primary template at a concentration of 2 ng/µL. Specific primer sets were designed for each candidate (Table S2), with the reverse primers conjugated to a Cy5 fluorophore to facilitate downstream cellular assays. To ensure high specificity and yield, gradient PCR was employed to determine the ideal annealing temperature based on the thermodynamic profiles of each primer pair. The reaction was performed in a 100 µL volume as shown in Table 6:

**Thermocycling conditions**:

- Initial denaturation 95^°^C for 3 min
- 30 Cycles:
  • Denaturation: 95°C for 30 s
  • Annealing: 50^°^C for 30 s
  • Extension: 72°C for 30 s
- Final extension: 72°C for 5 min

#### 4.8.2 Amplification From Commercial Template

The finalized candidates were synthesized from ANSA as purified DNA fragments and were received as lyophilized material. These candidates were then resuspended in nuclease‐free water at a stock concentration of 100 nM, and that stock was further diluted to 10 nM using nuclease‐free water. This template was then used for amplification using UMI‐specific primers. The reaction setup for finalized candidates is shown in Table 7:

**Thermal Cycling Conditions (Commercial)**:

- Initial Denaturation 95°C for 5 min
- 22 Cycles:
  • Denaturation: 95°C for 30 s
  • Annealing: 60°C for 30 s
  • Extension: 72°C for 30 s
- Final Extension: 72°C for 5 min

#### 4.8.3 Post‐Amplification Processing and Verification

PCR amplification efficiency and product integrity were assessed by agarose gel electrophoresis. Amplification products were separated on a 2% (*w*/*v*) agarose gel prepared in 1 × tris–borate–EDTA (TBE) buffer and stained with SYBR Safe DNA Gel Stain (Thermo Fisher) for nucleic acid visualization.

Gel imaging was performed using a Bio‐Rad Gel Doc documentation system to verify the presence of both the DNA backbone and the fluorophore label. DNA bands were visualized using standard SYBR Safe acquisition settings. Detection of Cy5‐labeled primers and their corresponding DNA products was achieved by operating the system in blot mode, with simultaneous signal acquisition in the AF488 (SYBR Safe) and Cy5 channels. Dual‐channel imaging enabled confirmation of incorporation of Cy5‐labeled primers in the amplified structures.

Following PCR, all candidates were purified using AMPure XP magnetic beads to remove unincorporated primers and reaction artifacts. To generate ssDNA or specific structural intermediates, the purified products were subjected to lambda exonuclease digestion. Subsequently, the candidates were refolded in 1 × T4 DNA ligase buffer to ensure proper structural assembly.

### 4.9 Flow Cytometry

Cells were seeded in 12‐well tissue culture plates one day prior to experimentation and maintained under standard culture conditions (37°C, 5% CO_2_) to ensure optimal adherence and recovery. On the day of stimulation, the culture medium was replaced with fresh serum‐free medium containing DNA nanostructures at a final concentration of 25 nM and incubated for 1 h. Following incubation, cells were washed with 1 × PBS, detached via trypsinization, and transferred to U‐bottom 96‐well plates. Each experimental condition was performed in biological duplicates or triplicates. To eliminate unbound nanostructures, cells were washed three times with 1 × PBS by centrifugation at 800 rpm for 5 min.

The cell pellets were subsequently fixed with 2% paraformaldehyde (PFA) for 10–15 min at room temperature, washed twice with 1 × PBS, and resuspended in FACS staining buffer (1 × PBS supplemented with serum). Samples were stored at 4°C protected from light and analyzed the same day. Untreated cells were included as negative controls to determine baseline fluorescence.

Flow cytometry data were acquired using a BD FACSCanto II system equipped with BD FACSDiva software, with 10 000 events recorded per sample. Data analysis and visualization were performed using FlowJo software employing conventional gating strategies.

### 4.10 Confocal Microscopy

Fixed cells were imaged using a Leica Stellaris X confocal scanning laser microscope (LIA). Samples were visualized using a 63 × oil‐immersion objective or a 40 × glycerol objective, as specified in the figure legends. Fluorophores were excited using the following laser lines: Hoechst (405 nm), LysoTracker Green (488 nm), and Cy5‐labeled DNA candidates (633 nm). To ensure imaging accuracy and eliminate spectral crosstalk, images were acquired using sequential scanning mode. For each sample, 3 and 4 z‐stacks were captured to assess and ensure representative sampling. Representative images displayed in the figures are projections generated from several fused optical sections from the middle of the Z‐stack to clearly illustrate internal localization.

Image processing was performed using Fiji (ImageJ, NIH). Processing was limited to background subtraction and brightness/contrast adjustments applied uniformly across all images within a comparison set to ensure representative visualization of signal intensities.

### 4.11 Stability Assay Under Physiological Conditions

The stability of all selected candidates and the refolded library was assessed under two conditions: DMEM supplemented with 10% FBS, and serum‐free DMEM. Samples were prepared at a final DNA concentration of 25 nM and incubated at 37°C for 0, 15, and 30 min, and 1, 2, and 4 h in FBS‐containing media, while the serum‐free condition was evaluated at the 4‐h time point only. All incubations were performed at 37°C, and reactions were quenched by transferring samples to −20°C if immediate gel loading was not possible. The 0‐min sample was prepared by mixing directly with media and loading immediately without incubation. A DNA sample diluted in 1 × PBS served as a positive control.

To verify the source of background fluorescence in the Cy5 channel was indeed from FBS, DMEM supplemented with 10% FBS, FBS alone, and serum‐free DMEM were run independently on the gel. A bright fluorescence band in the Cy5 channel was observed in both FBS‐containing lanes but was absent in the serum‐free condition, confirming that the background signal originates from FBS components. As this interference was present even at the 0‐min time point, stability in FBS‐containing conditions was assessed by comparison to the 0‐min sample rather than the untreated control.

## 5 Statistics

Statistical analyses were performed using GraphPad Prism 10. Data are presented as mean ± standard deviation (SD). For quantitative comparisons of flow cytometry data, statistical significance was determined using ordinary one‐way ANOVA. A *p*‐value ≤ 0.05 was defined as the threshold for statistical significance. Regarding experimental design, procedures were not randomized, and investigators were not blinded to group allocation during data collection or outcome assessment. All error bars presented in figures represent the SD of at least 3 independent biological replicates or as mentioned in the figure.

## Author Contributions

**Anjali Rajwar**: conceptualization, investigation, methodology, visualization, validation, formal analysis, data curation, writing – original draft, and writing – review and editing. **Lisa Eichhorn**: writing – review and editing, investigation, validation, software, and formal analysis. **Jakub Palacka**: writing – review and editing, investigation, and validation. **Sandra Ly**: investigation, software, formal analysis, and writing – review and editing. **Erik Benson**: conceptualization, funding acquisition, writing – original draft, writing – review and editing, visualization, methodology, project administration, and supervision.

## Funding

Research was supported by a grant from Vetenskapsrådet to EB with project ID 2022‐04147_VR and a grant from the European Research Council with a grant agreement ID 101221483, as well as a grant from the European Innovation Council with a grant agreement ID 101184647.

## Use of Generative AI and AI‐Assisted Technologies in the Writing Process

Anthropic Claude Opus 4.8 was used in the preparation of Figures S4–S6 and S8. Claude Opus 5.0 was used for suggestions of proof edits in the final manuscript.

## Conflicts of Interest

EB is an inventor on a patent related to the construction and selection of random DNA nanostructures (SE2350763A). EB is a shareholder and scientific advisor to Cubase Bio AB.

## Supporting information




**Supporting File**: adma74852‐sup‐0001‐SuppMat.docx.

## Data Availability

The data that support the findings of this study are openly available in Scilifelab Figshare at https://doi.org/10.17044/scilifelab.31323787.
