# Supplementary material for: Iterative Selection of DNA Nanostructures for Cellular Uptake
Source: Adv Mater. 2026 Aug 31;38(59):e74852. doi: 10.1002/adma.74852 (PMC13689900; doi:10.1002/adma.74852)
Supplement: Supplementary file 1 [file ADMA-38-e74852-s001.docx]

Supporting Information

**Iterative selection of DNA nanostructures for cellular uptake**

*Anjali Rajwar^1^, Lisa Eichhorn^1^, Jakub Palacka^1^, Sandra Ly^1^, Erik Benson^1#^*

*^1^ Science for Life Laboratory (SciLifeLab), Department of Microbiology, Tumor and Cell Biology, Karolinska Institutet, Solna, Sweden*

\# Corresponding author Email: [erik.benson@ki.se](mailto:erik.benson@ki.se)

*Figure. S1: Preparation of DNA Library.* (A) Denaturing 10% PAGE (19:1 acrylamide:bisacrylamide) containing 7 M urea stained with SYBR Gold, showing annealed and ligated fragments forming the library. “Ctrl” denotes the control, and “Lib+ExoIII” is the library treated with exonuclease digestion, which removes partially unfolded or smaller fragments. (B) 2% agarose gel stained with SYBR Gold showing PCR amplification of the control library and Lib+ExoIII at different annealing temperatures. (C) 2% agarose gel stained with SYBR Gold showing lambda exonuclease digestion of the PCR-amplified and purified library. (D) AFM images of the amplified and purified DNA library. Scale bar: 100 nm.

***Figure. S2: Cell Lysate Optimization for Subsequent Selection.*** (A) 2% agarose gel images stained with SYBR safe showing purification of treated and non-treated (control) lysates and their PCR amplification. (B) SYBR Safe–stained 2% agarose gel showing PCR efficiency at different amplification cycles. (C) Representative SYBR Safe–stained 2% agarose gel showing purified Round-6 lysate, its amplification, λ-exonuclease digestion, and refolding for the next round of cell stimulation.

***Figure S3:*  *Alignment of RAW264.7 sequencing reads to the mouse reference genome.*** RAW264.7 is a murine cell line, reads obtained from ONT sequencing were aligned to the mouse reference genome (GRCm39) using Minimap2 to determine the fraction of reads originating from genomic contamination. Crude denotes the initial library (R0) used for the first round of cell stimulation.

***Figure S4: Fragment diversity of the initial library from ONT data.*** A) Histogram of the number of unique fragment types in each read B) Histogram of the number of reads per unique fragment combination. Claude Opus 4.8 was used to aid with data extraction and plotting.

***Figure S5: Analysis of fragment combination enrichment and depletion across selection.*** Fragment combinations exhibiting the greatest increase or decrease in occurrence between the initial library and the round 10 selected libraries in HEK293T (A) and RAW264.7 (B), as determined by ONT sequencing. Analysis is restricted to combinations detectable in both the initial and selected library sequencing data.

Claude Opus 4.8 was used to aid with data extraction and plotting.

*Abbreviations and nomenclature: L, linear strand; KL, kissing loop; 3WJ, three-way junction; 4WJ, four-way junction; Hp, hairpin.* *The number following the structural abbreviation indicate the relevant length in nucleotides (nt), and the final number denotes strand identity. For example, L10.2 and L15.1 denote 10-nt linear strand 2 and 15-nt linear strand 1, respectively.*

*For bulged strands, the number before b indicates bulge size (e.g., L5b.1, 5-nt bulge, strand 1; L2b.1, 2-nt bulge, strand 1).*

***Figure S6: Fragment combination in initial and selected ONT data.*** After aligning the fragment sequences to the structure genomes throughout each selection round including the initial library, the composition of each read was evaluated and compared to the theoretical read composition given the occurrence of a certain fragment. For all initial and selected ONT data in HEK293T, RAW264.7 and Crude control, we compared the fraction of structure genomes where all expected oligonucleotides of all present fragments have been incorporated (all fragments paired), at least one fragment present had all matching oligonucleotides incorporated (some fragments paired), no fragments present had all their expected oligonucleotides incorporated or the read had no fragments present expect primer sequences (only primer). Claude Opus 4.8 was used to aid with data extraction and plotting.

***Figure S7: Additional analysis of UMI data*.** A) Histogram of occurrence of individual UMI’s in the sequencing data of the initial library from Illumina (top) and ONT (bottom), shows that most sequenced UMI’s occur only 1 time in the initial library. B) ONT data showing distribution of abundance, genome size (excluding primer sites) and length variability of UMI labelled structure genomes after 10 rounds of selection in two cell lines.

***Figure S8: Sequence identity of structure genomes that share the same UMI.*** Illumina data from round 10 in HEK293T was used to analyze the similarity between structure genomes that share a UMI and structure genomes with different UMIs. After filtering out sequences with high genomic similarity, UMIs with at least 50 reads were randomly selected to generate a consensus sequence based on all reads per UMI cluster. For each UMI cluster, a random subset of the reads was used against their corresponding consensus sequences to calculate the pairwise identity between the reads and the consensus sequence within a UMI cluster (left). To understand the similarity of structure genomes from different UMI clusters, 900 random instances of the pairwise identity between the consensus sequences that do not share UMI were calculated (right). Claude Opus 4.8 was used to aid with plotting.

***Figure S9: Empty selection and sequencing control*.** The Lib+ExoIII sample was amplified for 15 PCR cycles and purified using AMPure beads. This amplification and purification step was repeated for ten rounds without λ exonuclease digestion or refolding to assess amplification bias. The initial DNA nanostructure library was PCR amplified 10 times to understand the amplification preferences. The resulting products after 5 and 10 rounds of amplification were sequenced with Oxford nanopore. (A) Distribution of genome lengths after 5 or 10 rounds of amplification. (B) Structure fragment preference after 5 or 10 rounds of amplification compared to the crude library.

***Figure S10:*** ***Optimization of candidates selected from HEK293T nanopore sequencing of R-5.*** (A)Enrichment profile of selected candidates H5A-H5D across successive selection rounds from R-4 to R-10 expressed as fraction of total sequencing reads. (B) SYBR Safe–stained 2% agarose gel showing amplification efficiency of selected candidates across temperature gradient. (C) Cy5 channel imaging of gel in panel-B to confirm incorporation of the Cy5-labeled primer. (D) SYBR Safe–stained 2% agarose gel showing amplification of candidates at optimized annealing temperature and their subsequent purification. (E) Cy5 channel imaging of gel in panel-D confirming removal of unincorporated primers after purification.

*Abbreviations: Tpl is Template only, (+): PCR Amplification and (++): PCR amplification followed by Ampure purification*

***Figure S11:* *Genome mapping and alignment analysis of selected structural candidates.*** *(*A) BLAT analysis summary displaying the top five alignment hits for candidates H5A–H5D against the human reference genome (GRCh38). Higher scores indicate greater sequence homology between the candidate query and the host genome. (B) Detailed alignment maps of the primary hit for each candidate. The matching bases between the query and genomic sequences are indicated by capitalized blue letters. Light blue highlights mark the boundaries of gaps or transitions (such as potential splice sites) within the sequence alignment and black non capitalized letters mark no significant alignments and is mostly on the 3 ' end of the query sequence.

***Figure S12:*** ***Verification of genomic origin in control samples.*** Agarose gels stained with SYBR Safe were imaged using the blot setting of a gel documentation system, with AF488 displayed in green and Cy5 in red. (A) Initial library (pre-selection library) showing all candidates with a faint H5D band, and empty selection R-5, representing the initial library subjected to multiple rounds of amplification and AMPure purification without cell stimulation. (B) Empty selection R-10 showing faint bands for H5A and H5B, with no detectable H5C and H5D. (C) Amplification from non-treated HEK control cells to assess cellular contamination, showing a band only for H5A and very faint H5B.

*Abbreviations: Initial Lib: initial library before any cell stimulation, Initial Lib+: PCR amplified initial lib using amplification loop Cy5 primers, ES-R5 Tpl: Empty selection library after five rounds of amplification and purification used as template, ES-R10: Empty selection library after ten round of amplification and purification used as template, Ctrl Tpl: HEK293T non-treated control purified lysate used as template.*

***Figure S13: Cellular uptake of selected candidates in HEK293T cells.*** (A) representative laser scanning confocal images of fixed cells stimulated with 10 nM of individual candidates amplified from the Round-5 template. (B) Cellular uptake of candidates amplified from Round-10 template and control samples. Preselection library (initial lib) was the library used for the initial round of cell stimulation and Round-10 library (R10-Lib) was the library after ten rounds of selection were controls amplified using Cy5-labeled UMI primers. Nuclei are stained with Hoechst (blue), and Cy5-labeled DNA candidates are shown in red; the merged panel displays combined blue and red channels. Scale bar: 10 µm

***Figure S14: Assessment of potential false-positive signals from Cy5-labeled primer mixtures.*** Confocal microscopy images were acquired to monitor the cellular uptake of residual primers from UMI and H5B sets. Primers were pooled and added to cells at a final concentration of 50 nM to evaluate whether unreacted primer mixtures contribute to false-positive intracellular signals. The red channel represents the Cy5 signal, the blue channel represents Hoechst-stained nuclei, and the merged panel shows the overlay of both channels. Scale bar: 20 μm.

***Figure S15: Optimization of HEK293T and RAW264.7 selected candidates from R-10 Illumina data****.* (A) 2% agarose gel stained with SYBR safe demonstrating the purity of the constructs that will be used as template for subsequent amplification. (B) PCR purified, λ-exo digested, and refolded RAW264.7 selected candidates. Agarose gel stained with SYBR safe and imaged in blot mode; with green color indicates the SYBR stained DNA and red color indicate Cy5 primer incorporation. (C) PCR purified, λ-exo digested, and refolded HEK293T selected candidates under same conditions as in (B). All the candidates showed successful amplification without any non-specific amplification.

*Abbreviations: P stands for PCR purified, λ: λ-exo digested and RF stands for Refolded*

***Figure S16: Optimization of control candidates for cellular study.*** **(A)** 2% Agarose gel electrophoresis of unselected control candidates (US-A-D) visualized using SYBR Safe (green; AF488 channel) and Cy5 fluorescence (red). Lane designations indicate: "Amplified" (unselected control PCR product), "Purified" (samples after AMPure bead purification showing removal of residual primers), and "Template" (stock template loaded to assess initial heterogeneity). **(B)** SYBR Safe-stained agarose gel of newly selected random candidates (Lanes 1–9) and control candidates H5A (Lane 10) and H5D (Lane 11). **(C)** PCR amplification of random candidates from template shown in panel B using Cy5-labeled primers, visualized via SYBR Safe (green; AF488) and Cy5 (red) channels. Highlighted lanes indicate the specific candidates selected for further optimization and cellular study. **(D)** Temperature optimization of selected random control candidates; NTC denotes the No Template Control.

***Figure S17: Agarose gel verification of finalized candidates*.**  Amplified candidates were resolved on an agarose gel to verify assembly and purity. Samples were loaded as indicated on the respective lanes and electrophoresed for 45 minutes at 100V. Lane L denotes the 100 bp DNA ladder. The green signal represents SYBR Safe DNA stain acquired under the AF488 channel, while the red signal represents Cy5 detection.

***Figure S18: Fragment composition of the selected DNA nanostructure candidates.*** Each candidate is represented by its constituent sequence elements, showing the contribution of fragments derived from the initial library. Fragment types are color coded; linear fragments in green, linear bulged fragments in dark green, 3-way junctions in blue, 4-way junctions in orange, hairpins in red, kissing-loops in magenta, primers from the amplification loop in dark grey and candidate primers (primers added during candidate synthesization) in light grey. Fragment specifics are denoted along each structure genome with the following abbreviations; Rev: reverse, Fwd: forward, rc: reverse complement, ed: edit distance. For instance, does H5D include the following; 'Cand Fwd ed0': Candidate forward primer mapped to structure genome with edit distance 0 based on fragment mapping algorithm, magenta '14 nt S1 rc ed7': Kissing loop with length 14 nt strand 1 but as the reverse complement (rc) of the sequence for KL-14nt-Strand1 in Table S1, 'Cand Rev rc ed0': Candidate reverse primer as its reverse complement with edit distance 0.

***Figure S19: In Silico Folding Analysis and secondary structure prediction of Finalized DNA Sequences predicted by m-Fold***

***Figure S20 oxDNA simulation of candidate structures.*** *First,* the minimum-free-energy secondary structure of each sequence was simulated using NUPACK 4 (DNA model, 37 °C, 1 µM strand concentration, 1.0 M Na⁺), producing a dot-bracket structure showing the predicted base-pairing. This was converted to an oxDNA input 3D coarse-grained geometry with idealized B-form helical stems, with ‘mutual trap’ simulation forces between nucleotides predicted to base pair. Each structure was relaxed using a sequence of two oxDNA simulations (DNA model, 1.0 M salt), a Monte Carlo energy minimization (1 × 10⁵ steps, 30 °C) to resolve overlaps, followed by GPU-accelerated molecular dynamics (1 × 10⁶ steps, 20 °C, dt = 0.003) with the base-pair restraints applied throughout, yielding a final relaxed 3D structure that was visualized with oxVIEW.

***Figure S21:*** ***Quantification of DNA nanostructure internalization via flow cytometry in RAW264.7 cells.*** Data shown represent the gated Cy5-high cell population. **(A)** Representative flow cytometry scatter plots of RAW264.7 cells treated with DNA nanostructure candidates selected from HEK293T screens. **(B)** Quantification of Cy+ cells calculated from scatter plots shown in (A). **(C)** Representative flow cytometry scatter plots of cells treated with candidates selected from RAW264.7-specific screens. **(D)** Quantification of Cy+ cells calculated from scatter plots shown in (C). **(E)** Representative flow cytometry scatter plots of cells treated with randomly selected candidates. **(F)** Quantification of Cy+ cells calculated from scatter plots shown in (E). For all flow cytometry experiments, 10,000 events were recorded per sample. Samples were acquired in quadruplets (n = 4), and data represent two independent experiments (N = 2). Error bars indicate mean ± SD. Statistical significance was determined by ordinary one-way ANOVA (****P < 0.0001, ns: non-significant).

***Figure S22:*** ***Quantification of DNA nanostructure internalization via flow cytometry in A549 cells.*** **(A)** Representative flow cytometry scatter plots of A549 cells treated with DNA nanostructure candidates selected from HEK293T screens. **(B)** Quantification of Cy+ cells calculated from scatter plots shown in (A). **(C)** Representative flow cytometry scatter plots with candidates selected from RAW264.7 specific screens. **(D)** Quantification of Cy+ cells calculated from scatter plots shown in (C). **(E)** Representative flow cytometry scatter plots of cells treated with randomly selected candidates. **(F)** Quantification of Cy+ cells calculated from scatter plots shown in (E). For all flow cytometry experiments, 10,000 events were recorded per sample. Samples were acquired in duplicate (n = 2). The experiment was performed independently twice (N = 2) with similar results; representative data from one experiment are presented. Error bars indicate mean ± SD. Statistical significance was determined by ordinary one-way ANOVA (****P < 0.0001,***P < 0.0005, **P < 0.0012, ns: non-significant).

***Figure S23:***  ***Quantification of mean fluorescence intensity (MFI) calculated from corresponding flow cytometry dataset.*** MFI for the finalized DNA nanostructure candidates in HEK293T (A), RAW264.7 (B), and A549 (C) cells. MFI values represent the average Cy5 fluorescence signal per cell, providing an additional measure of nanostructure uptake beyond the percentage of Cy5-positive cells. Error bars indicate mean ± SD. Statistical significance was determined by ordinary one-way ANOVA (****P < 0.0001,***P < 0.0005, **P < 0.0012, ns: non-significant).

‘

***Figure S24: Qualitative assessment of cellular uptake of final candidates in RAW264.7 using laser scanning confocal microscopy.*** **(A)** Confocal images of cells treated with HEK293T-selected DNA nanostructure (DN-Cy5). **(B)** Confocal images of cells treated with RAW cell-selected DNA nanostructure. **(C)** Confocal images of RAW264.7 cells treated with randomly selected DNA nanostructure. For all panels, the red channel represents DN-Cy5 uptake, and the bottom panels represent merged images of all channels with nuclei stained with Hoechst dye (blue). Scale bar: 20 μm.

***Figure S25: Qualitative assessment of cellular uptake of final candidates in A549 cells using confocal microscopy.*** **(A)** Confocal images of A549 cells treated with HEK293T-selected DNA nanostructure (DN-Cy5). **(B)** Confocal images of RAW cell-selected candidates. **(C)** Confocal images of randomly selected candidates. For all panels, the red channel represents DN-Cy5 uptake, the green channel indicates LysoTracker Green staining for lysosomes, and the bottom panels represent merged images of all channels with nuclei stained with Hoechst dye (blue). Scale bar: 15 μm.

***Figure S26: Stability assessment of selected DNA nanostructure candidates in biological media.*** Representative 2% agarose gel electrophoresis analysis showing the stability of Cy5-labeled DNA nanostructure candidates incubated in DMEM supplemented with 10% FBS complete media (CM) for 0 min, 30 min, 1 hr, 2 hr, and 4 hr with stability test extended to 6 hours for HEK293T-selected candidates. PBS was included as a control condition. We also tested the stability in serum-free DMEM (SF) for 4 h to mimic the selection conditions. Gel with controls without DNs containing SF, CM, and FBS alone were included. The same incubation conditions and time points were tested for all candidate nanostructures. Samples were loaded as mentioned in the respective lanes and electrophoresed for 45 mins at 150V. Gels were imaged using BioRad ChemiDoc imager with green channel (SYBR Safe staining) and red channel (Cy5-labeled nanostructures). A prominent signal observed in the Cy5 channel for FBS-containing samples originates from serum-associated background fluorescence. L denotes the 100 bp DNA ladder.

***Figure S27: Qualitative assessment of cellular uptake of final candidates in MDA-MB-231 cells using confocal microscopy.*** **(A)** Confocal images of MDA-MB-231 cells treated with HEK293T-selected DNA nanostructure (DN-Cy5). **(B)** Confocal images of RAW cell-selected candidates. **(C)** Confocal images of randomly selected candidates. For all panels, the red channel represents DN-Cy5 uptake, and the bottom panels represent merged images of all channels with nuclei stained with Hoechst dye (blue). Scale bar: 15 μm.

***Figure S28:*** ***Quantification of DNA nanostructure internalization via flow cytometry in MCF-7 cells.*** **(A)** Representative flow cytometry scatter plots of MCF-7 cells treated with finalized DNA nanostructure candidates. **(B)** Quantification of Cy+ cells calculated from scatter plots shown in (A). **(C)** Quantification of mean fluorescence intensity of Cy5+ cells under live cell population. For this experiment, 10,000 events were recorded per sample. Samples were acquired in duplicate (n = 2). Error bars indicate mean ± SD. Statistical significance was determined by ordinary one-way ANOVA (****P < 0.0001,***P < 0.0005, **P < 0.005, *P < 0.05, ns: non-significant).

‘

***Figure S29:*** ***Quantification of DNA nanostructure internalization via flow cytometry in HeLa cells.*** **(A)** Representative flow cytometry scatter plots of HeLa cells treated with finalized DNA nanostructure candidates. **(B)** Quantification of Cy5+ cells calculated from scatter plots shown in (A). **(C)** Quantification of mean fluorescence intensity of Cy5+ cells under live cell population. For this experiment, 10,000 events were recorded per sample. Samples were acquired in triplicate (n = 3). The experiment was performed independently twice (N = 2) with similar results; representative data from one experiment are presented. Error bars indicate mean ± SD. Statistical significance was determined by ordinary one-way ANOVA (****P < 0.0001,***P < 0.0005, **P < 0.005, *P < 0.05 ns: non-significant).

## ***Table S1: Sequence information of individual fragments used for library preparation***

| **Name** | | **Sequence** |
| --- | --- | --- |
| 3WJ Strand1 | | /5Phos/ACTCTCTGTATGTAGCATACGCGCACG |
| 3WJ Strand2 | | /5Phos/AGTCGTGCGCGTATGCGTAGCTGCGAG |
| 3WJ Strand3 | | /5Phos/ACTCTCGCAGCTACGCTACATACAGAG |
| 3WJ unpairedT Strand1 | | /5Phos/ACTCTGCACGTGTGGTCGTGTATCAGCG |
| 3WJ unpairedT Strand2 | | /5Phos/AGTCGCTGATACACGTCCGATGATACGG |
| 3WJ unpairedT Strand3 | | /5Phos/ACTCCGTATCATCGGTCCACACGTGCAG |
| 4WJ Strand1 | | /5Phos/ACTCGCTATCTACGGCCGCATGATACG |
| 4WJ Strand2 | | /5Phos/AGTCGTATCATGCGGCGCATATCATGG |
| 4WJ Strand3 | | /5Phos/ACTCCATGATATGCGCCTGTGCATGAG |
| 4WJ Strand4 | | /5Phos/ACTCTCATGCACAGGCCGTAGATAGCG |
| KL-10nt Strand1 | | /5Phos/AGTCCATCATGTGCAGGCATGCGCTACCCTGCACATGATGG |
| KL-10nt Strand2 | | /5Phos/AGTCTACATGATACGTGGTAGCGCATGCACGTATCATGTAG |
| KL-12nt-Strand1 | | /5Phos/AGTCTATCTGTACATGGTGCTATGATATACCATGTACAGATAG |
| KL-12nt-Strand2 | | /5Phos/AGTCTGTATCACACGCGTGCTATGATATACGCGTGTGATACAG |
| KL-14nt-Strand1 | | /5Phos/AGTCCTATACACAGCAGTGTGCATATGTATGCTGCTGTGTATAGG |
| KL-14nt-Strand2 | | /5Phos/AGTCTATACACACGCTGCATACATATGCACACAGCGTGTGTATAG |
| KL-16nt-Strand1 | | /5Phos/AGTCACATATATCTGCGCGTAGATAGCTGCTGACGCAGATATATGTG |
| KL-16nt-Strand2 | | /5Phos/AGTCATCATATACGCGGTCAGCAGCTATCTACGCCGCGTATATGATG |
| KL-18nt-Strand1 | | /5Phos/AGTCACGTAGCGCACAGGATACAGCAGCTATGTAGCTGTGCGCTACGTG |
| KL-18nt-Strand2 | | /5Phos/AGTCTGTGCACAGATAGCTACATAGCTGCTGTATCCTATCTGTGCACAG |
| KL-20nt-Strand1 | | /5Phos/AGTCTATCGATAGCTGGTCTACAGATACATGCGCGTACCAGCTATCGATAG |
| KL-20nt-Strand2 | | /5Phos/AGTCCTATACAGATAGGTACGCGCATGTATCTGTAGACCTATCTGTATAGG |
| Amplification Loop | | /5Phos/ ACTCCATACGTATCTANNNNNNNNNNACATATACATATCACGTGCTGGCCTACACGCGTACATGTATAGANNNNNNNNNNTAGATACGTATGG |
| Hairpin 10nt | | /5Phos/AGTCGCACACGATACAGACACACATGACTGTATCGTGTGC |
| Hairpin 12nt | | /5Phos/AGTCCGCATCATATGTACGCATCAGATCATATGATGCGG |
| Hairpin 14nt | | /5Phos/AGTCATATCGATATGTGACATATCATCGCGTCACATATCGATATG |
| Hairpin 16nt | | /5Phos/AGTCCATCTGTGTGCGGTGCGCATGATATAGATCCGCACACAGATGG |
| Hairpin 18nt | | /5Phos/AGTCCTATCGATATATGCAGATATCGTATCGCAGCCATATATCGATAGG |
| Linear 10nt Strand1 | | /5Phos/AGTCACGTGATACGG |
| Linear 10nt Strand2 | | /5Phos/ACTCCGTATCACGTG |
| Linear 11nt Strand1 | | /5Phos/AGTCATACGCGTATAG |
| Linear 11nt Strand2 | | /5Phos/ACTCTATACGCGTATG |
| Linear 12nt Strand1 | | /5Phos/AGTCCGCAGCGTACGAG |
| Linear 12nt Strand2 | | /5Phos/ACTCTCGTACGCTGCGG |
| Linear 13nt Strand1 | | /5Phos/AGTCCATCATCACATATG |
|  | Linear 13nt Strand2 | /5Phos/ACTCATATGTGATGATGG |
|  | Linear 14nt Strand1 | /5Phos/AGTCATCAGCTGCATGTGG |
|  | Linear 14nt Strand2 | /5Phos/ACTCCACATGCAGCTGATG |
|  | Linear 15nt Strand1 | /5Phos/AGTCCTGCATCTATCGCTAG |
|  | Linear 15nt Strand2 | /5Phos/ACTCTAGCGATAGATGCAGG |
|  | Linear 16nt Strand1 | /5Phos/AGTCGATATATAGCGCTGCGG |
|  | Linear 16nt Strand2 | /5Phos/ACTCCGCAGCGCTATATATCG |
|  | Linear 17nt Strand1 | /5Phos/AGTCTATCTGCTAGCTGTGTGG |
|  | Linear 17nt Strand2 | /5Phos/ACTCCACACAGCTAGCAGATAG |
|  | Linear 18nt Strand1 | /5Phos/AGTCTAGCTACGCGTATATCGTG |
|  | Linear 18nt Strand2 | /5Phos/ACTCACGATATACGCGTAGCTAG |
|  | Linear 19nt Strand1 | /5Phos/AGTCCGCTGATATCTACACACGCG |
|  | Linear 19nt Strand2 | /5Phos/ACTCGCGTGTGTAGATATCAGCGG |
|  | Linear 20nt Strand1 | /5Phos/AGTCTCTATCGCGCAGCTATCACAG |
|  | Linear 20nt Strand2 | /5Phos/ACTCTGTGATAGCTGCGCGATAGAG |
|  | Linear 1nt bulge Strand1 | /5Phos/AGTCTATGTGCGATGCTATCATGTG |
|  | Linear 1nt bulge Strand2 | /5Phos/ACTCACATGATAGCACGCACATAG |
|  | Linear 2nt bulge Strand1 | /5Phos/AGTCATCATGATACGTGTACGCTAG |
|  | Linear 2nt bulge Strand2 | /5Phos/ACTCTAGCGTACACGTCATGATG |
|  | Linear 3nt bulge Strand1 | /5Phos/AGTCCGATATATACGTAGCACGCAG |
|  | Linear 3nt bulge Strand2 | /5Phos/ACTCTGCGTGCTACTATATCGG |
|  | Linear 4nt bulge Strand1 | /5Phos/AGTCCATCACATACAGCGTAGCTAG |
|  | Linear 4nt bulge Strand2 | /5Phos/ACTCTAGCTACGCTGTGATGG |
|  | Linear 5nt bulge Strand1 | /5Phos/AGTCATATCACATATCGCACGTAGG |
|  | Linear 5nt bulge Strand2 | /5Phos/ACTCCTACGTGTGTGATATG |
|  | Forward Primer | /5Phos/CCATATGTGTACACGTGTGATA |
|  | Reverse Primer | CCTGCGCAGCTATATCTATCAG |

## ***Table S2: Cluster Primer Sequences***

| Name | Sequence |
| --- | --- |
| H5A_FWD | /5Phos/ATGTATATGTTAACGAAGAA |
| H5A_BWD | /5Cy5/CATGTATAGACAAGCGATAA |
| H5B_FWD | /5Phos/ATGTATATGTGGACTTCAGG |
| H5B_BWD | /5Cy5/CATGTATAGACGCCGATTGG |
| H5C_FWD | /5Phos/ATGTATATGTATCTCCCAGA |
| H5C_BWD | /5Cy5/CATGTATAGATATTGGCATC |
| H5D_FWD | /5Phos/ATGTATATGTTGACCTTACC |
| H5D_BWD | /5Cy5/CATGTATAGAGGAGTGAGAG |
| STD_FWD | /5Phos/CCAGCACGTGATATGTATATGT |
| STD_BWD | /5Cy5/CCTACACGCGTACATGTATAGA |

***Table S3: UMI counts of candidates.*** Fraction of reads in each sequencing dataset (not filtered to remove biological data) that contains the fused UMI of the candidates used in this study. Pairwise alignment was used to also find UMI’s that may contain read errors (primarily in ONT data).

| **Candidate Name** |  |  |  |  |  |  |  |  |  |  |  |  |  |  |
| --- | --- | --- | --- | --- | --- | --- | --- | --- | --- | --- | --- | --- | --- | --- |
| Cell lines | N/A | HEK293T | HEK293T | HEK293T | HEK293T | RAW264.7 | RAW264.7 | RAW264.7 | RAW264.7 | N/A | HEK293T | HEK293T | RAW264.7 | RAW264.7 |
| Round | **0** | **4** | **6** | **8** | **10** | **4** | **6** | **8** | **10** | **0** | **5** | **10** | **5** | **10** |
| Sequencing method | **ONT** | **ONT** | **ONT** | **ONT** | **ONT** | **ONT** | **ONT** | **ONT** | **ONT** | **Illumina** | **Illumina** | **Illumina** | **Illumina** | **Illumina** |
| H5A | 0,00E+00 | 8,98E-05 | 1,94E-01 | 4,01E-01 | 4,67E-01 | 1,65E-03 | 9,31E-03 | 5,26E-02 | 8,70E-03 | 5,78E-05 | 1,19E-06 | 2,17E-01 | 7,32E-04 | 3,09E-03 |
| H5B | 0,00E+00 | 1,26E-05 | 1,78E-03 | 1,82E-03 | 9,87E-04 | 0,00E+00 | 0,00E+00 | 1,85E-05 | 1,44E-05 | 2,75E-07 | 0,00E+00 | 8,98E-05 | 1,85E-07 | 2,36E-06 |
| H5C | 0,00E+00 | 3,59E-06 | 1,77E-03 | 3,65E-03 | 5,65E-03 | 1,91E-06 | 0,00E+00 | 4,63E-06 | 0,00E+00 | 1,37E-07 | 0,00E+00 | 1,96E-03 | 3,69E-07 | 0,00E+00 |
| H5D | 0,00E+00 | 1,80E-06 | 1,85E-04 | 5,16E-04 | 4,62E-04 | 0,00E+00 | 0,00E+00 | 0,00E+00 | 1,20E-06 | 4,12E-07 | 4,76E-07 | 7,00E-05 | 1,85E-07 | 3,37E-07 |
| H10A | 0,00E+00 | 1,80E-06 | 4,04E-05 | 9,66E-05 | 1,92E-04 | 0,00E+00 | 0,00E+00 | 0,00E+00 | 1,20E-06 | 1,37E-07 | 2,38E-07 | 6,77E-04 | 1,85E-07 | 1,35E-06 |
| H10B | 0,00E+00 | 1,80E-06 | 9,81E-05 | 1,53E-04 | 1,18E-04 | 0,00E+00 | 0,00E+00 | 0,00E+00 | 0,00E+00 | 1,37E-07 | 4,76E-07 | 4,12E-04 | 1,85E-07 | 1,01E-06 |
| H10C | 0,00E+00 | 0,00E+00 | 6,06E-05 | 6,18E-05 | 6,93E-05 | 0,00E+00 | 0,00E+00 | 0,00E+00 | 0,00E+00 | 2,75E-07 | 0,00E+00 | 1,10E-04 | 0,00E+00 | 0,00E+00 |
| H10D | 0,00E+00 | 1,39E-03 | 3,75E-03 | 3,83E-03 | 3,16E-03 | 5,98E-03 | 7,70E-03 | 8,93E-03 | 1,18E-02 | 2,95E-05 | 1,27E-04 | 5,36E-03 | 7,28E-03 | 1,35E-02 |
| R10A | 0,00E+00 | 1,03E-03 | 2,88E-03 | 3,19E-03 | 2,61E-03 | 4,95E-03 | 6,11E-03 | 7,31E-03 | 9,35E-03 | 2,25E-05 | 1,11E-04 | 4,54E-03 | 6,28E-03 | 1,16E-02 |
| R10B | 0,00E+00 | 1,36E-03 | 3,41E-03 | 3,55E-03 | 2,96E-03 | 6,20E-03 | 8,09E-03 | 9,33E-03 | 1,18E-02 | 3,23E-05 | 1,38E-04 | 5,21E-03 | 8,07E-03 | 1,42E-02 |
| R10C | 0,00E+00 | 3,82E-04 | 8,89E-04 | 5,99E-04 | 3,87E-04 | 1,45E-03 | 2,22E-03 | 2,40E-03 | 3,22E-03 | 6,45E-06 | 9,16E-04 | 9,03E-04 | 3,48E-03 | 6,19E-03 |
| R10D | 0,00E+00 | 8,74E-04 | 2,02E-03 | 1,80E-03 | 1,43E-03 | 4,06E-03 | 4,93E-03 | 5,66E-03 | 7,25E-03 | 1,52E-05 | 8,85E-05 | 2,44E-03 | 4,96E-03 | 8,73E-03 |
| Random-1 | 0,00E+00 | 0,00E+00 | 0,00E+00 | 0,00E+00 | 0,00E+00 | 1,91E-06 | 0,00E+00 | 0,00E+00 | 3,61E-06 | 5,49E-07 | 0,00E+00 | 0,00E+00 | 0,00E+00 | 5,05E-07 |
| Random-2 | 0,00E+00 | 0,00E+00 | 0,00E+00 | 0,00E+00 | 0,00E+00 | 0,00E+00 | 0,00E+00 | 0,00E+00 | 0,00E+00 | 1,37E-07 | 0,00E+00 | 0,00E+00 | 0,00E+00 | 0,00E+00 |
| Random-3 | 0,00E+00 | 0,00E+00 | 0,00E+00 | 0,00E+00 | 0,00E+00 | 0,00E+00 | 0,00E+00 | 0,00E+00 | 0,00E+00 | 1,37E-07 | 2,38E-07 | 0,00E+00 | 0,00E+00 | 0,00E+00 |
| Random-4 | 0,00E+00 | 0,00E+00 | 0,00E+00 | 0,00E+00 | 0,00E+00 | 0,00E+00 | 0,00E+00 | 0,00E+00 | 0,00E+00 | 9,61E-07 | 4,76E-07 | 0,00E+00 | 3,69E-07 | 0,00E+00 |
| Random-5 | 0,00E+00 | 0,00E+00 | 0,00E+00 | 0,00E+00 | 0,00E+00 | 0,00E+00 | 0,00E+00 | 0,00E+00 | 0,00E+00 | 4,12E-07 | 0,00E+00 | 0,00E+00 | 0,00E+00 | 0,00E+00 |
| Random-6 | 0,00E+00 | 0,00E+00 | 0,00E+00 | 0,00E+00 | 0,00E+00 | 0,00E+00 | 0,00E+00 | 0,00E+00 | 0,00E+00 | 1,37E-07 | 0,00E+00 | 0,00E+00 | 0,00E+00 | 0,00E+00 |
| Random-7 | 0,00E+00 | 0,00E+00 | 0,00E+00 | 0,00E+00 | 0,00E+00 | 0,00E+00 | 0,00E+00 | 0,00E+00 | 0,00E+00 | 1,37E-07 | 0,00E+00 | 0,00E+00 | 0,00E+00 | 0,00E+00 |
| Random-8 | 0,00E+00 | 0,00E+00 | 0,00E+00 | 0,00E+00 | 0,00E+00 | 0,00E+00 | 0,00E+00 | 0,00E+00 | 0,00E+00 | 2,75E-07 | 0,00E+00 | 0,00E+00 | 0,00E+00 | 0,00E+00 |
| Random-9 | 0,00E+00 | 0,00E+00 | 0,00E+00 | 0,00E+00 | 0,00E+00 | 0,00E+00 | 0,00E+00 | 0,00E+00 | 0,00E+00 | 5,49E-07 | 0,00E+00 | 0,00E+00 | 0,00E+00 | 0,00E+00 |
| US-A | 0,00E+00 | 0,00E+00 | 0,00E+00 | 0,00E+00 | 0,00E+00 | 0,00E+00 | 0,00E+00 | 0,00E+00 | 0,00E+00 | 1,37E-07 | 0,00E+00 | 0,00E+00 | 0,00E+00 | 0,00E+00 |
| US-B | 0,00E+00 | 0,00E+00 | 0,00E+00 | 0,00E+00 | 0,00E+00 | 0,00E+00 | 0,00E+00 | 0,00E+00 | 0,00E+00 | 1,37E-07 | 0,00E+00 | 0,00E+00 | 0,00E+00 | 0,00E+00 |
| US-C | 0,00E+00 | 0,00E+00 | 0,00E+00 | 0,00E+00 | 0,00E+00 | 0,00E+00 | 0,00E+00 | 0,00E+00 | 0,00E+00 | 1,37E-07 | 0,00E+00 | 0,00E+00 | 0,00E+00 | 3,37E-07 |
| US-D | 0,00E+00 | 0,00E+00 | 0,00E+00 | 0,00E+00 | 0,00E+00 | 0,00E+00 | 0,00E+00 | 0,00E+00 | 0,00E+00 | 1,37E-07 | 0,00E+00 | 0,00E+00 | 0,00E+00 | 0,00E+00 |

***Table S4: Finalized candidates and their corresponding sequence information***.

| **Name** | **Sequence** |
| --- | --- |
| R10A | CCATATGTGTACACGTGTGATAACGCGTGTAGGCCAGCACGTGATATGTATATGCCTCCACATGCAGCTGATGACTCTGTGATAGCTGCGCGATAGAGACTCTAGCTACGCTGTATGTGATGGACTCCGCACAGCTAGCAGATAGACTCATATGTGATGATGGACTCTAGCTACGCTGTATGTGATGGACTCCATGATATGCGCCGCATGATACGACTCCATACGTATCTAGCTCCCCCCCCTGATAGATATAGCTGCGGAGG |
| R10B | CCATATGTGTACACGTGTGATAACGCGTGTAGGCCGGCACGTGATATGTATATGTTATGTTTACCTAGATACGTATGGAGTCCATCACAGCGTAGCTAGAGTCTATGTGCGTGCTATCATGTGAGTCCTATATAGGTCTACAGATACATGCGCGTACCTATCTGTATAGGACTCCTACGTGCTATACGTGATATGACTCCGTATCATCGGACGTGTATCAGCGACTCCTACGTGCGATATGTGATATGACTCCATACGTATCTACCCCGCCCCCCTGATAGATATAGCTGCGGAGG |
| R10C | CCATATGTGTACACGTGTGATAACGCGTGTAGGCCAGCACGCGATATGTATATGTCACGCTCGTCTAGGTACGTATGATCTGATGCGTACATATGATGCGGACTCCACACAGCTAGCAGACAGACTCCACACAGCTAGCAGATAGACTCGCGTGTGTAGATATCAGCGGACTCTCGCAGCTACGTATACGCGCACGACTCCGTATCACGTGACTCACGATATACGCGTAGCTAGACTCCACATGCAGCTGATGACTCCTACGTGCGATATGTGATATGACTCCATACGTATCTACCCTACCTCTTCTATATATGTACGCGTGTAGGCCAGCACGTGATATGTATACGTTGCTTCTTCCTAGATACGTATGGAACTGATAGATATAGCTGCGGAGG |
| R10D | CCATATGTGTACACGTGTGATAACGCGTGTAGGGCAGCACGTGATATGTATATGTGACAGCACCATAGATACGTATGGAGTCCTATCGATATATGGCTGCGATACGATATCTGCATATATCGATAGGACTCTAGCGTACACGATCATGATGACTCCACATGCAGCTGATGACTCCACATGCAGCTGATGACTCCACATGCAGCTGATGACTCCATACGTATCTACCCCTCTCCGCTGATAGATATAGCTGCGGAGG |
| H10A- | CCATATGTGTACACGTGTGATAGGTTGCCAAATAGATACGTATGGAGTCACGTGATACGGAGTCTGTGCACAGATAGGATACAGCAGCTAGGACATAAGCTCTATACATGTACGTGTGTAGGCCAGCACGTGATATGTATATGTGGTTGCCAAATAGATACGTATGGAGTCACGTGATACGGAGTCTGTGCACAGATAGGATACAGCAGCTAGGACATAAGCCTGATAGATATAGCTGCGGAGG |
| H10B | CCATATGTGTACACGTGTGATAGAGGTGACGGGTATCATCGGACGTGTATCAGCGACTCCGTATTATCGGACGTGTATCAGCGACTCCATACGTATCTACAGTGAGTGGTCTATACATGTACGCGTGTAGGCCAGCACGTGATATGTATATGTGAGGCGACGGGTATCATCGGACGTGTATCAGCGACTCCGTATCATCGGACGCGTATCAGCGACTCCATACGTATCTACAGTGAGTGGCTGATAGATATAGCTGCGGAGG |
| H10C | CCATATGTGTACACGTGTGATACAAACAAACTTAAATACGTACAAATAAACTCCACATGCAGCTAATAACTCACAACATACGCGTAGCTAAACTCCATACGTATCTAACAGGAACGCTCTATACATGTACGCCGTGGAGGCCAGCACGTGATATGTATGTGTCAAACAAACTTAAATACGTACAAATAGACTCCACATGCAGCTGATGACTCACGATATACGCGTAGCTAGACTCCATACGTATCCAACAGGAACGC-CTGATAGATATAGCTGCGGAGG |
| H10D | CCATATGTGTACACGTGTGATAACGCGTGTAGGCCAGCACGTGATATGTATATGTTAAAGGGCATTAGATACGTATGGAGTCCATCACAGCGTAGCTAGAGTCATCAGCTGCATGTGGAGTCCATCATCACATATGAGTCATCATATACGCGGCGTAGATAGCCGCTGACCGCGTATATGATGACTCTATACGCGTATGACTCCTATGTGCGATATGTGATATGACTCCATACGTATCTACCCCCCCCC**-**CTGATAGATATAGCTGCGGAGG |
| Unselected  Control C | CCATATGTGTACACGTGTGATAATACTAATCCTAGATACGTATGGAGTCCGCAGCGTACGAGAGTCCATCATGTGCAGGGTAGCGCATGCCTGCACATGATGGACTCGCTATCTACGGCCTGTGCATGAGAGTCTATGTGCGTGCTGTCATGTGAGTCATATCGATATGTGACGCGATGATATGTCACATATCGATATGACTCCATGATAGCATCGCACATAGACTCTCATGCACAGGCGCATATCGTGGAGTCTGTGCACAGATAGGATACAGCAGCTGATAGATATAGCTGCGCAGG |
| Random-1 | CCATATGTGTACACGTGTGATACCCACGAAAATAGATACGTATGGAGTCATCTATACATGTACGCGTGTAGGCCAGCACGTGATATGTATATGTTCATTAAGATCTAGATACGTATGGAGTCACATATATCTGCGTCAGCAGCTATCTACGCGCAGATATATGTGACTCCATACGTATCTAATCTCCCGCTCTGATAGATATAGCTGCGCAGG |
| Random-4 | CCATATGTGTACACGTGTGATAACGCGTGTAGGCCAGCACGTGATATGTATATGTTAGACATACCTAGATACGTATGGAGTCCATCATCACATATGAGTCCGATATAGTAGCACGCAGAGTCCTATACACAGCAGCATACATATGCACACTGCTGTGTATAGGACTCTGCGTGCTACGTATATATCGGACTCATATGTGATGATGGACTCCATACGTATCTACCGGCCGCTCCTGATAGATATAGCTGCGCAGG |
| Random-6 | CCATATGTGTACACGTGTGATATTGCTTACATTAGATACGTATGGAGTCCTATACAGATAGGTCTACAGATACATGCGCGTACCTATCTGTATAGGACTCCATACGTATCTATATGGTGCCTTCTATACATGTACGCGTGTAGGCCAGCACGTGATATGTATATGTTTGCTTACATTAGATACGTATGGAGTCCTATACAGATAGGTCTACAGATACATGCGCGTACCTATCTGTATAGGACCCCATACGTATCTATATGGTGCCTCTGATAGATATAGCTGCGCAGG |
| H5B | CCATATGTGTACACGTGTGATAGGACTTCAGGAGTGGAACCACAGACCTTCACAGTGAGTGTTACAGCTCTTAAAGGTGGCGCGTCCGGAGTTGTTTGTTCCTCTCGGTGGGTTTGTGGTCTCACTGACTTCAGGAATGGAGTCTAGACCCTCGCAGTGAGTGTTACAGCTCATAAAAGTAGTGTGGACCCAAAGAGTGACCATCAGCAAGATTTATTGTGAAGAGCGAAAGAACAAAGCTTCCACAGTATGGAAGGGGACCCACAGCTAGCGGATAGACTCCAGGACGCACTCACCAATTGGCGCTGATAGATATAGCTGCGCAGG |
| H5D | CCATATGTGTACACGTGTGATATGACCTTACCATACCTCATATGTTCAATTAACACACTATATTTTAAAACAGACTCAGATCTTGAAAACAAGATATAAGGTCCAATCCCTATACATGGTGCGTAAGGTGGCTTTGGGGAATACAGTAGAAACCTAGTTTTCAGCATGGCTCTCAGAAAACACCATCCCTCCTGCCTGTGGTAAGTATTCGTAGTAAGAGCTGAAACGGTGGGGTCAAGAGAACATCACTTCGGGTGAGAAAGTTTGTTTCATACTTTATTTCTCACAATTCTCGCCGTCAAGAGAGGAGTTTGATTCATCAACTTTGGGATGCATTAGAATAACCCGTGGGATGGATTAAATAGATTCCATGGCCCTGCTCCTGAGAGATCCTTATTCAGTGGATGTGACGTGCGACTTAACTATCACTTAGCTTAATTTACTTCACAGTCAGTGTACATATGCACACTGCTGTGTATAGAACTCCATACGTATCTACTCACTCCCTGATAGATATAGCTGCGCAGG |

***Table S5: Structural characteristics of finalized candidates based on mfold secondary structure predictions***

| **Sequence Name** | **Length (nt)** | **Energy (ΔG)** | **Tracebacks** | **Helix Count** | **Stability Density (ΔG/nt)** |
| --- | --- | --- | --- | --- | --- |
| Random-4 | 254 | -85.7 | 1 | 107 | 0.337 |
| Random-6 | 288 | -61.5 | 3 | 116 | 0.214 |
| Unselected control-C | 233 | -61.5 | 1 | 37 | 0.331 |
| H10D | 271 | -47.5 | 2 | 104 | 0.175 |
| R10B | 296 | -44.1 | 2 | 60 | 0.149 |
| R10D | 256 | -39.9 | 1 | 74 | 0.156 |
| Random-1 | 213 | -39.9 | 2 | 49 | 0.187 |
| H10A | 244 | -21.0 | 3+ | 38 | 0.086 |
| H5D | 528 | -28.5 | 1 | 116 | 0.054 |
| R10C | 395 | -42.1 | 3 | 152 | 0.107 |
| R10A | 263 | -26.2 | 2 | 41 | 0.099 |
| H10B | 262 | -25.4 | 3 | 58 | 0.090 |
| H5B | 327 | -25.8 | 2 | 44 | 0.079 |
| H10C | 279 | -20.8 | 3 | 51 | 0.075 |
